# Homozygous *SGCB* splice-site variant causes isolated dilated cardiomyopathy through sarcoglycan complex destabilization in East Asians

**DOI:** 10.1172/JCI198675

**Published:** 2026-06-01

**Authors:** Fangfang Li, Haruki Shinomiya, Yuki Kuramoto, Koshiro Kanaoka, Yuji Sakahashi, Yasuki Ishihara, Hidetaka Kioka, Seiko Ide, Yumi Yamaguchi-Kabata, Shu Tadaka, Ikuko N. Motoike, Kengo Kinoshita, Kinuko Ohneda, Hidetoshi Sakurai, Takahiro Okumura, Yohei Miyashita, Kota Jojima, Hisakazu Kato, Ken Matsuoka, Kazuya Tanabe, Shunsuke Nishimura, Seiji Takashima, Yoshihiro Asano, Yasushi Sakata

**Affiliations:** 1 Department of Cardiovascular Medicine, The University of Osaka Graduate School of Medicine, Osaka, Japan.; 2 Department of Genomic Medicine and; 3 Department of Pathophysiology of Heart Failure and Therapeutics, National Cerebral and Cardiovascular Center Research Institute, Osaka, Japan.; 4 Premium Research Institute for Human Metaverse Medicine (WPI-PRIMe), The University of Osaka, Osaka, Japan.; 5 Department of Medical and Health Information Management, National Cerebral and Cardiovascular Center Research Institute, Osaka, Japan.; 6 Omics Research Center and; 7 Biobank, National Cerebral and Cardiovascular Center, Osaka, Japan.; 8 Department of Dental Anesthesiology, The University of Osaka Graduate School of Dentistry, Osaka, Japan.; 9 Tohoku Medical Megabank Organization,; 10 Graduate School of Information Sciences, and; 11 Advanced Research Center for Innovations in Next-Generation Medicine, Tohoku University, Sendai, Japan.; 12 Department of Clinical Application, Center for iPS Cell Research and Application (CiRA), Kyoto University, Kyoto, Japan.; 13 Department of Cardiology and; 14 Department of Advanced Cardiovascular Therapeutics, Nagoya University Graduate School of Medicine, Nagoya, Japan.; 15 Department of Medical Biochemistry, The University of Osaka Graduate School of Medicine, Osaka, Japan.; 16 Department of Medical Biochemistry, The University of Osaka Graduate School of Frontier Biosciences, Osaka, Japan.

**Keywords:** Cardiology, Genetics, Cardiovascular disease, Genetic diseases

## Abstract

Dilated cardiomyopathy (DCM) is a genetically heterogeneous disorder, characterized by ventricular dilatation and impaired systolic function, leading to heart failure and sudden cardiac death. Despite advances in genomic technologies, the genetic cause of DCM remains unidentified in more than half of the cases. Here, we performed an integrative analysis of genomic and transcriptomic data from patient-derived cardiac tissue to identify causative variants in genetically undiagnosed DCM. This approach enabled us to identify a homozygous splice-site variant (c.243+6T>A) in the sarcoglycan gene *SGCB*, which results in exon 2 skipping. This variant was significantly enriched in patients with DCM compared with the general population, with consistent genotype–phenotype correlations observed across multiple families. Protein-level analysis of cardiac tissue from homozygous individuals revealed loss of β-sarcoglycan, the protein product of *SGCB*, and destabilization of the sarcoglycan complex. Although *SGCB* has been previously associated with limb-girdle muscular dystrophy, these homozygous individuals showed no biochemical or clinical signs of skeletal muscle involvement, indicating an absence of myopathy. Compared with variant-negative patients with DCM, homozygous individuals also had a higher risk of early-onset adverse cardiac events. Together, these findings identify c.243+6T>A in *SGCB* as a cause of isolated DCM associated with unfavorable clinical outcomes.

## Abstract 2

An exon-skipping SGCB variant causes isolated dilated cardiomyopathy without skeletal muscle involvement by destabilizing the sarcoglycan complex, resulting in early-onset adverse cardiac events.

## Graphical abstract

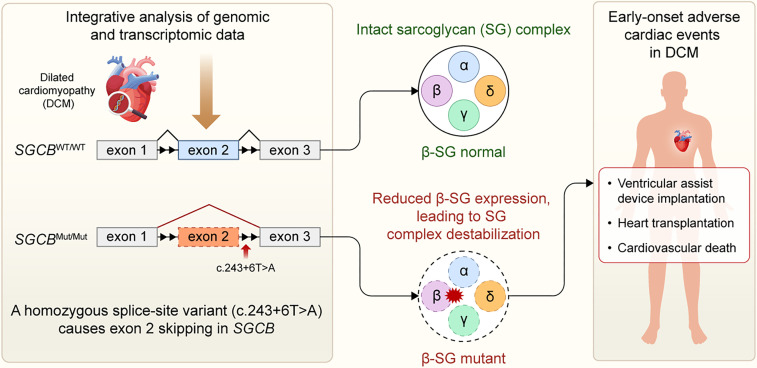

## Introduction

Dilated cardiomyopathy (DCM) is a myocardial disease characterized by ventricular dilatation and impaired systolic function in the absence of hypertensive, valvular, congenital, or ischemic heart disease (1). As the disease progresses, patients with DCM often suffer heart failure and occasionally sudden cardiac death. DCM is a genetically heterogeneous disorder, with more than 30 disease-associated genes identified to date (2, 3). Recent studies have demonstrated that pathogenic variants can be identified in approximately 40% of DCM cases; however, in more than half of the patients, the underlying genetic cause remains unknown (3, 4). Although DCM is one of the most common indications for heart transplantation (HTx), and significant progress has been made in the development of novel devices and pharmacologic agents, a definitive curative treatment has yet to be established (5). One major barrier to therapeutic development is the incomplete understanding of its genetic and molecular basis.

Recent advances in DNA-based technologies, such as whole-exome sequencing (WES) and whole-genome sequencing (WGS), have substantially increased the identification of genetic variants, including those in noncoding regions (6). However, the functional interpretation of these variants remains a major bottleneck in genetic diagnosis. To address this challenge, RNA-seq has recently been adopted as a complementary approach to DNA-based analyses (7, 8). RNA-seq enables the detection of transcriptomic abnormalities, such as aberrant expression, aberrant splicing, and monoallelic expression (9, 10). Several studies have shown that integrating RNA-seq with DNA sequencing can improve diagnostic yield by about 7.5%–36% (7, 8). Despite its utility, comprehensive transcriptome studies using patient-derived cardiac tissue remain limited, largely due to the difficulty in obtaining such samples.

The *SGCB* gene encodes β-sarcoglycan (β-SG), a component of the sarcoglycan complex located in the cell membrane. This complex consists of 4 sarcoglycan subunits — α, β, γ, and δ — and plays a crucial role in maintaining the structural linkage between the cytoskeleton and the extracellular matrix, mainly in cardiac and skeletal muscle cells (11). Pathogenic homozygous or compound heterozygous variants in any of the 4 sarcoglycan genes are known to cause limb-girdle muscular dystrophy (LGMD) (12). While many patients with LGMD develop concomitant DCM, *SGCB* has not previously been clearly implicated as a cause of isolated DCM.

Here, we applied an integrative approach combining DNA sequencing and RNA-seq data from patient-derived cardiac tissues to identify causative variants in genetically undiagnosed cases of DCM. This approach enabled us to identify a homozygous splice-site variant (c.243+6T>A) in *SGCB* as the likely causal variant. Furthermore, protein-level analysis of the cardiac tissue revealed that this *SGCB* variant destabilizes the sarcoglycan complex, supporting its pathogenic role in the development of DCM.

## Results

### Identification of SGCB as a splicing and expression outlier in genetically undiagnosed DCM.

To identify potential causative genes in genetically undiagnosed patients with DCM, we applied the Detection of RNA Outliers Pipeline (DROP), a previously published framework for detecting aberrant gene expression and splicing events (10), to RNA-seq data from cardiac tissues. Cardiac tissue samples for the DROP analysis were obtained from 107 patients who underwent HTx or ventricular assist device (VAD) implantation, including 63 with DCM and 44 with other cardiac conditions. Inclusion of the latter group enabled exclusion of transcriptomic changes commonly associated with end-stage heart failure and enhanced the statistical power of the analysis. Among the 63 DCM patients, WES identified pathogenic or likely pathogenic (P/LP) variants in 37 (59%) across 40 known DCM-associated genes (Supplemental Table 1; supplemental material available online with this article; https://doi.org/10.1172/JCI198675DS1), based on American College of Medical Genetics and Genomics (ACMG) criteria. The remaining 26 (41%) were genetically undiagnosed. We thus focused on these undiagnosed cases within the results of the DROP analysis and progressively narrowed down candidate genes after screening all genes for transcriptomic abnormalities (Supplemental Figure 1).

**Figure 1:**
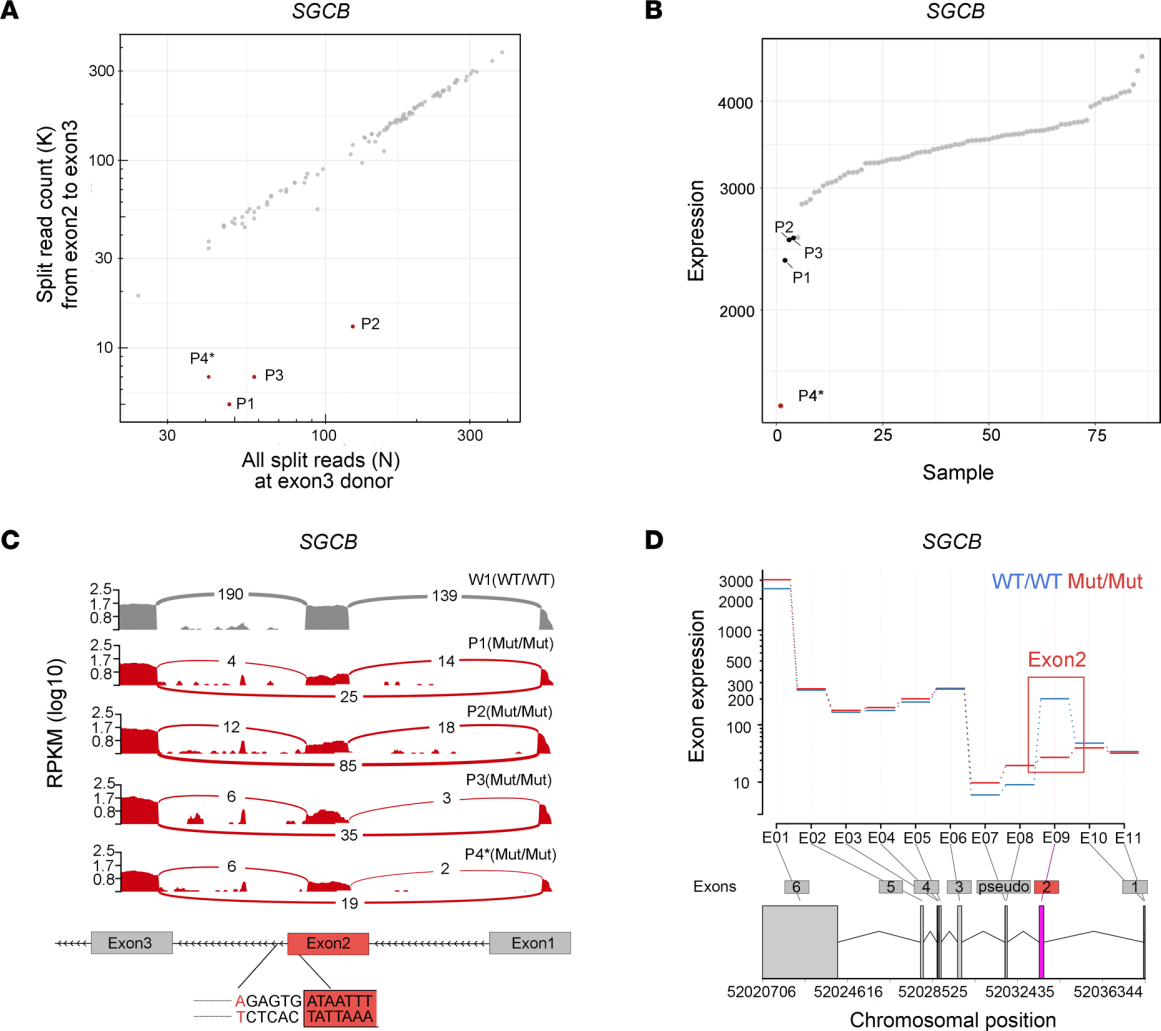
Aberrant splicing and altered expression of *SGCB* in cardiac tissue from patients with DCM. (**A**) Splicing rank plot showing aberrant splicing of *SGCB*. (**B**) Expression rank plot demonstrating decreased expression of *SGCB*. (**C**) Sashimi plots illustrating splicing patterns across exons 1–3 in samples carrying either the WT (WT/WT) or homozygous c.243+6T>A (Mut/Mut) *SGCB* allele, identified through DROP analysis. RPKM, reads per kilobase of transcript per million mapped reads. (**D**) Differential exon usage analysis using DEXSeq comparing WT/WT and Mut/Mut groups across all annotated *SGCB* exons (exons 1–6) and a pseudoexon located between exons 2 and 3. Each bar represents normalized exon-level read counts. The *x* axis represents exon analysis numbers assigned by DEXSeq, with canonical *SGCB* exon numbers annotated below to facilitate alignment with the annotated gene structure; corresponding chromosomal positions are also displayed below. Exon 2, which is significantly decreased expression in the Mut/Mut group, is highlighted in red. P4* denotes a patient harboring 2 heterozygous variants: c.243+6T>A and c.325C>T (p.Arg109*).

In the splicing outlier analysis, aberrant splicing events were identified in 1,203 genes among the genetically undiagnosed DCM cases. Filtering for genes carrying rare variants detected by WGS that were predicted to cause splicing defects, followed by exclusion of genes with low cardiac expression, resulted in 13 genes. Of these, *SGCB* was the only gene identified in multiple cases (Supplemental Figure 1 and P1–P4 in Figure 1A).

In the expression outlier analysis, we focused on genes with markedly reduced expression (fold change ≤ 0.5) and identified 299 candidate genes. Filtering for rare variants detected by WGS with potential regulatory effects, followed by exclusion of genes with low cardiac expression, resulted in a single candidate gene, *SGCB* (Figure 1B and Supplemental Figure 1). Notably, P4, the case exhibiting this aberrant expression of *SGCB*, was also identified as a splicing outlier (Figure 1A).

### The c.243+6T>A splice-site variant causes exon 2 skipping in SGCB.

All 4 cases (P1–P4) with splicing abnormalities in *SGCB* shared the common splicing event: exon 2 skipping (Figure 1C). WGS revealed the same splice-site variant (*SGCB* c.243+6T>A) in intron 2 in all 4 cases: P1–P3 were homozygous, and P4 was heterozygous. Sanger sequencing additionally confirmed the variant in these cases (Supplemental Figure 2). Importantly, P4, the case identified as both a splicing and expression outlier, also harbored a nonsense variant (c.325C>T, p.Arg109*; Supplemental Figure 2).

To quantitatively assess exon-level expression, we performed differential exon usage analysis using the Bioconductor package DEXSeq, a previously described method for detecting exon-specific expression changes from RNA-seq data (13). This analysis revealed that among exons 1–6 of *SGCB*, only exon 2 exhibited significantly reduced expression in homozygous c.243+6T>A (Mut/Mut) samples compared with WT (WT/WT) (Figure 1D), consistent with exon 2 skipping. PCR analysis using cDNA derived from cardiac tissue confirmed that the exon 2–retained transcript (i.e., the nonskipped transcript) showed a c.243+6T>A genotype–dependent stepwise decrease from WT/WT to WT/Mut and Mut/Mut, whereas the exon 2–skipped transcript showed a corresponding stepwise increase (Supplemental Figure 3). Notably, in the Mut/Mut samples, an aberrant transcript containing a cryptic exon was also detected (Supplemental Figure 3), which, unlike the in-frame exon 2 skipping, introduced a premature stop codon within the cryptic exon (Supplemental Figure 4). Digital PCR (dPCR) analysis further provided quantitative validation, demonstrating a progressive reduction of exon 2 expression across genotypes (Supplemental Figure 5). These results indicate that the c.243+6T>A variant induces exon 2 skipping in an allele dose–dependent manner.

### The homozygous SGCB c.243+6T>A variant is significantly enriched in patients with DCM.

To further assess the pathogenicity of the *SGCB* c.243+6T>A variant associated with exon 2 skipping, we compared its allele frequency and zygosity between the general population and DCM patient cohorts. The minor allele frequency (MAF) of this variant was 0.0003 in the general global population (gnomAD v4.1.0, all populations) and 0.009 in East Asian populations (gnomAD v4.1.0, East Asian), indicating a notable population-specific enrichment. We thus compared carrier frequencies between a population-matched general Japanese cohort (Tohoku Medical Megabank Organization [ToMMo], *n* = 54,212) and a Japanese DCM cohort (*n* = 936). To formally address potential confounding by subtle ancestry differences between cohorts, we assessed genome-wide population structure by principal component analysis (PCA); the DCM cohort and ToMMo largely overlapped in PCA space, and DCM cases homozygous for the *SGCB* variant did not form a distinct ancestry cluster (Supplemental Figure 6). We then evaluated enrichment using logistic regression with principal components as covariates (3 ToMMo controls were excluded due to missing genotypes for this variant). In this PC-adjusted analysis, the heterozygous carriers were observed in 569/54,209 ToMMo controls (1.05%) and 13/936 DCM cases (1.39%). This difference did not reach conventional statistical significance (OR, 1.83; 95% CI, 0.99–3.38; *P* = 0.053). In contrast, the homozygous carriers were observed in 3/54,209 ToMMo controls (0.006%) and 10/936 DCM cases (1.07%). This enrichment was highly significant (OR, 227.55; 95% CI, 41.43–1,249.71; *P* = 4.23 × 10^–10^). These findings suggest that the heterozygous state of the c.243+6T>A variant is consistent with an asymptomatic carrier status for an autosomal recessive condition, whereas the homozygous state is markedly enriched among patients with DCM, supporting its pathogenic role.

### Genotype–phenotype concordance further supports the SGCB variant as a genetic cause of DCM.

**Figure 2:**
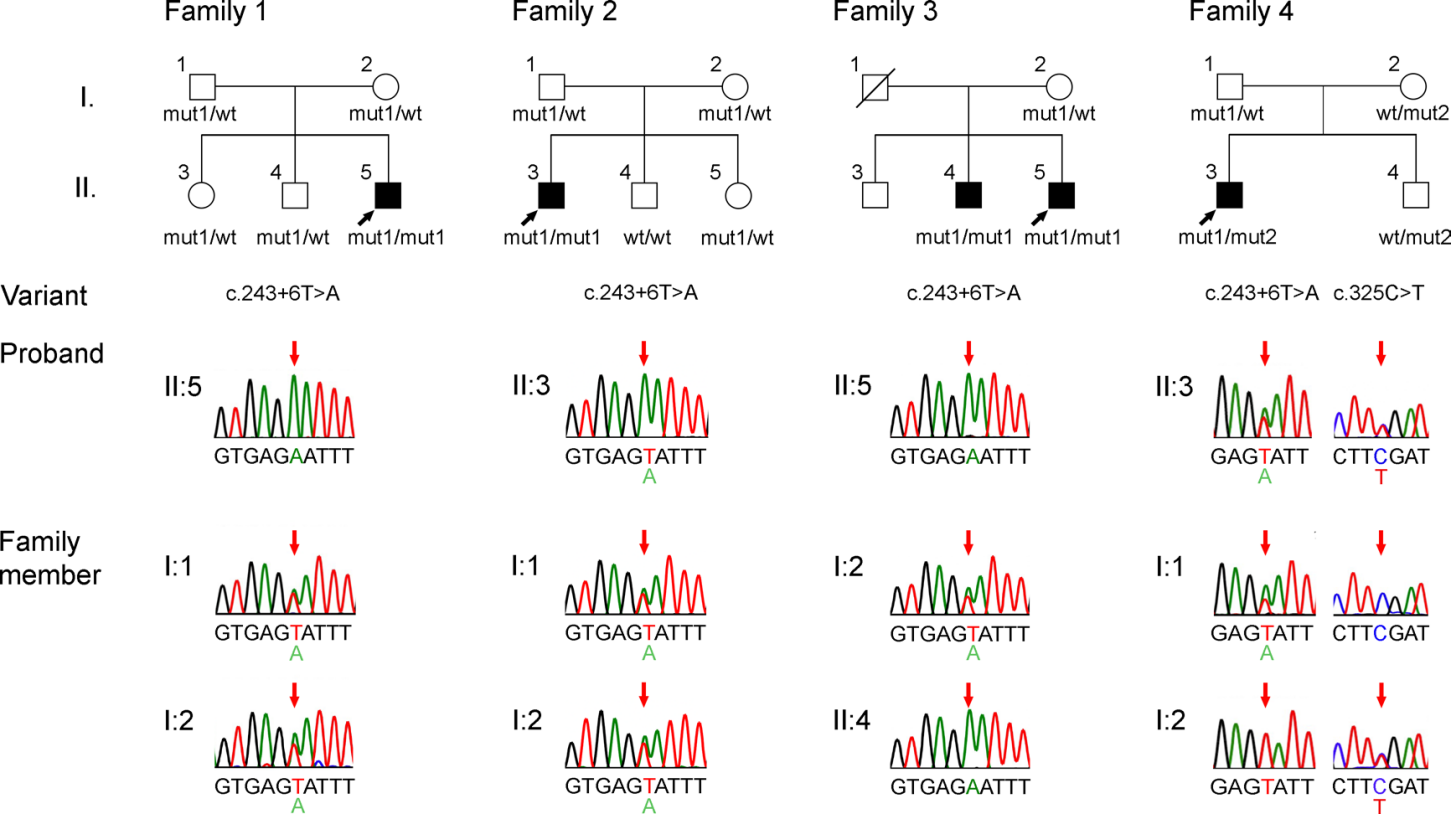
Pedigrees of 4 unrelated families carrying the *SGCB* c.243+6T>A variant. The proband in each family is indicated by a black arrow. Filled symbols, affected individuals; open symbols, unaffected; slashed open symbols, deceased unaffected individuals. Sanger sequencing chromatograms show the variant site marked with red arrows. wt, mut1, and mut2 indicate the WT allele at c.243+6, 243+6T>A, and c.325C>T, respectively. In family 4, the proband harbors compound heterozygous variants (mut1 and mut2).

A clear correlation between genotype and phenotype is a crucial criterion for determining the pathogenicity of a genetic variant (14). Thus, we collected clinical and genetic information from 4 pedigrees harboring the *SGCB* variant (Figure 2). Families 1 and 4 represent probands identified via transcriptomic outlier analysis (P2 and P4 in Figure 1A; P4 also shown in Figure 1B), while families 2 and 3 were independently extracted from the DCM database. In families 1–3, the probands with DCM were homozygous for the *SGCB* c.243+6T>A variant. In families 1 and 2, no other family members exhibited an apparent cardiac phenotype, and each of them was either WT or heterozygous for the variant. In family 3, not only the proband but also his brother developed severe DCM, and the brother was found to carry the same homozygous *SGCB* variant. In contrast, their mother, who showed no cardiac symptoms, was heterozygous for the variant. In family 4, the proband with DCM harbored the 2 heterozygous *SGCB* variants shown in Supplemental Figure 2 (P4). Notably, the proband’s father (I:1), who carried the heterozygous c.243+6T>A variant, and mother (I:2), who carried the heterozygous c.325C>T variant, exhibited no cardiac manifestations. These findings confirmed that the proband from family 4 was compound heterozygous for the 2 variants.

We also evaluated other genetic causes of DCM to exclude the possibility that the disease was attributable to alternative pathogenic variants. We screened all 12 cases carrying biallelic pathogenic *SGCB* variants identified in the DCM cohort, comprising 11 homozygous for c.243+6T>A and 1 compound heterozygous for c.243+6T>A and c.325C>T (p.Arg109*), for known P/LP variants in 40 DCM-associated genes (Supplemental Table 1). No such variants were identified in 10 of the 12 cases. Among the remaining 2, 1 carried a likely pathogenic variant in *TTN* (c.33580+2T>C, NM_001267550.2); however, due to the lack of familial information, its contribution to the disease could not be definitively determined. Another case (family 3, II:5) carried a heterozygous *DES* variant (c.1210A>G, NM_001927.4), but his affected sibling (family 3, II:4), who shared the homozygous *SGCB* c.243+6T>A variant*,* did not carry the *DES* variant. This intrafamilial segregation pattern implicates the *SGCB* variant as the primary cause of DCM in family 3. To further exclude structural variants as an alternative genetic cause, copy number variant (CNV) analysis was performed in the 10 cases with WGS data available; no reportable CNVs affecting the DCM-associated genes were identified.

### Exon 2 skipping of SGCB leads to loss of β-SG and destabilization of the sarcoglycan complex.

**Figure 3:**
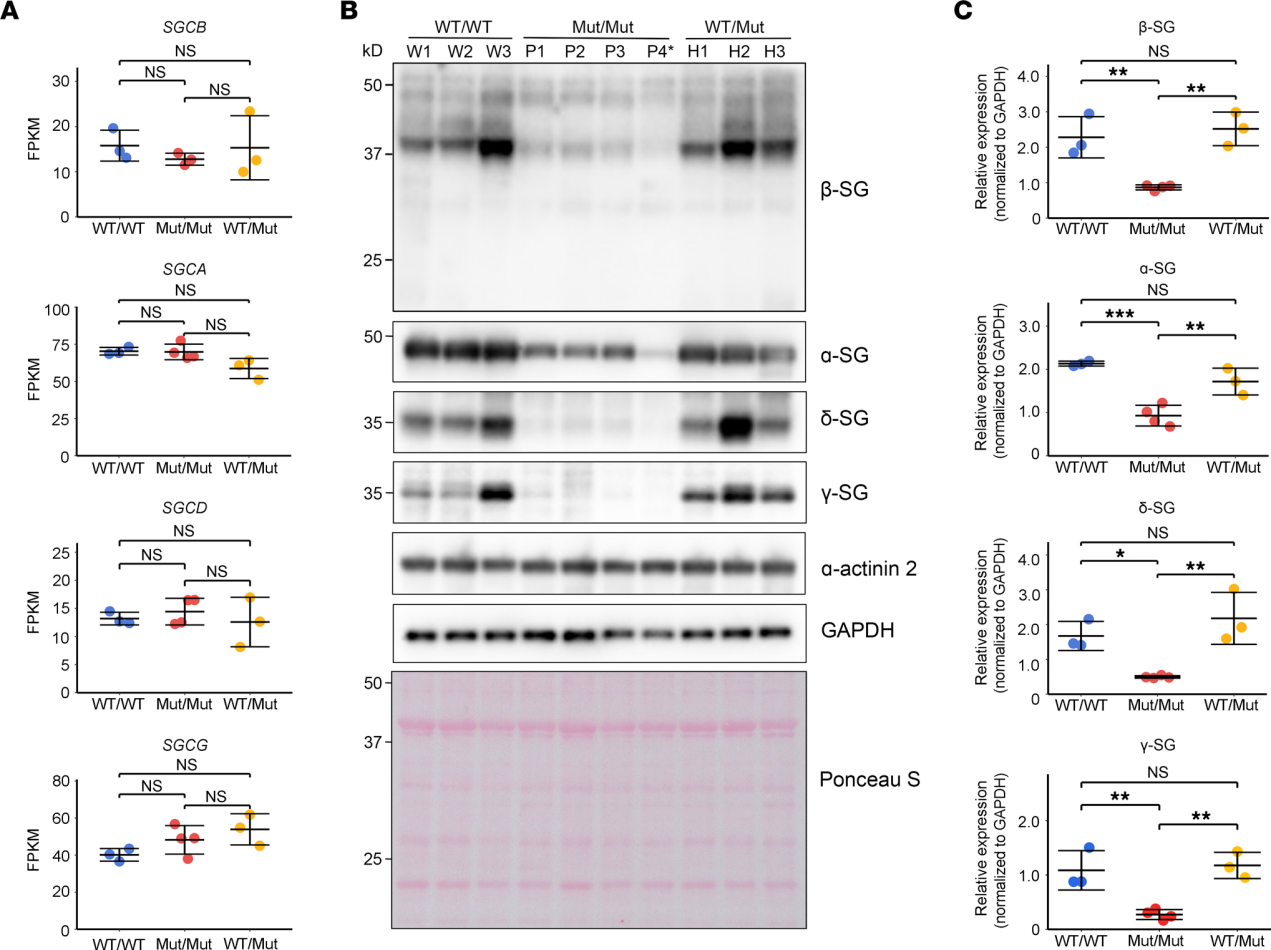
Gene and protein expression of sarcoglycan subunits in cardiac tissue. (**A**) RNA-seq–based expression levels of *SGCB*, *SGCA*, *SGCD*, and *SGCG* in cardiac tissue samples from individuals carrying the WT (WT/WT, *n* = 3), homozygous c.243+6T>A (Mut/Mut), or heterozygous c.243+6T>A (WT/Mut, *n* = 3) allele of *SGCB*. For *SGCB*, the Mut/Mut group includes 3 samples, excluding the compound heterozygous sample (P4), which was identified as an expression outlier in Figure 1B. For *SGCA*, *SGCD*, and *SGCG*, the Mut/Mut group includes 4 samples, including P4. Expression values, presented as fragments per kilobase of transcript per million mapped reads (FPKM), are shown as mean ± SD. (**B**) Representative Western blot analysis of β-SG, α-SG, δ-SG, and γ-SG in cardiac tissue samples from the same groups (WT/WT, *n* = 3; Mut/Mut, *n* = 4; WT/Mut, *n* = 2). α-Actinin 2, GAPDH, and Ponceau S staining were used as loading controls. P4* indicates a compound heterozygous sample harboring the c.243+6T>A and c.325C>T variants. (**C**) Quantification of Western blot band intensities shown in **B**, performed using ImageJ. Data are presented as the mean ± SD. *P < 0.05; **P < 0.01; ***P < 0.001. *P* values were calculated using 1-way ANOVA followed by the Tukey-Kramer test for pairwise comparisons.

*SGCB* encodes β-SG, a core component of the sarcoglycan complex. Given that the c.243+6T>A variant induces exon 2 skipping at the mRNA level, we sought to determine how this splicing alteration affects the expression and membrane localization of β-SG and other components of the sarcoglycan complex in patient cardiac tissue. β-SG is a single-pass transmembrane protein (15). In silico topology prediction indicated that the exon 2–skipped isoform lacks most of the predicted transmembrane segment, suggesting a marked disruption of the membrane-spanning region (Supplemental Figure 7). To test this, we performed Western blot analysis using an antibody capable of detecting the exon 2–skipped isoform. Although exon 2–skipped mRNA was readily detectable in patient samples (Supplemental Figure 3), the corresponding skipped β-SG protein isoform (~25 kDa) was undetectable (Figure 3B). This suggests that the exon-skipped protein is unstable and subject to rapid degradation. To further evaluate the stability of this isoform, we overexpressed it in cultured cells (Supplemental Figure 8, A and B). Although the skipped β-SG isoform was detectable, it appeared faint and smeared, indicating an increased susceptibility to proteolytic degradation.

In addition to the absence of the skipped isoform, full-length β-SG was markedly reduced in Mut/Mut samples compared with WT/WT and WT/Mut samples (Figure 3, B and C), indicating an overall loss-of-function effect of the variant. To determine whether this reduction affects other components of the sarcoglycan complex, we examined the expression of α-, γ-, and δ-SG in the same samples (Figure 3, B and C). All showed decreased expression, with α-SG showing a relatively modest reduction. Notably, the β-SG antibody did not cross-react with other sarcoglycan subunits (Supplemental Figure 8C). Because mRNA expression levels of each subunit were preserved in Mut/Mut samples (Figure 3A), the reduction in protein levels likely reflects posttranscriptional instability of the complex rather than transcriptional downregulation.

**Figure 4:**
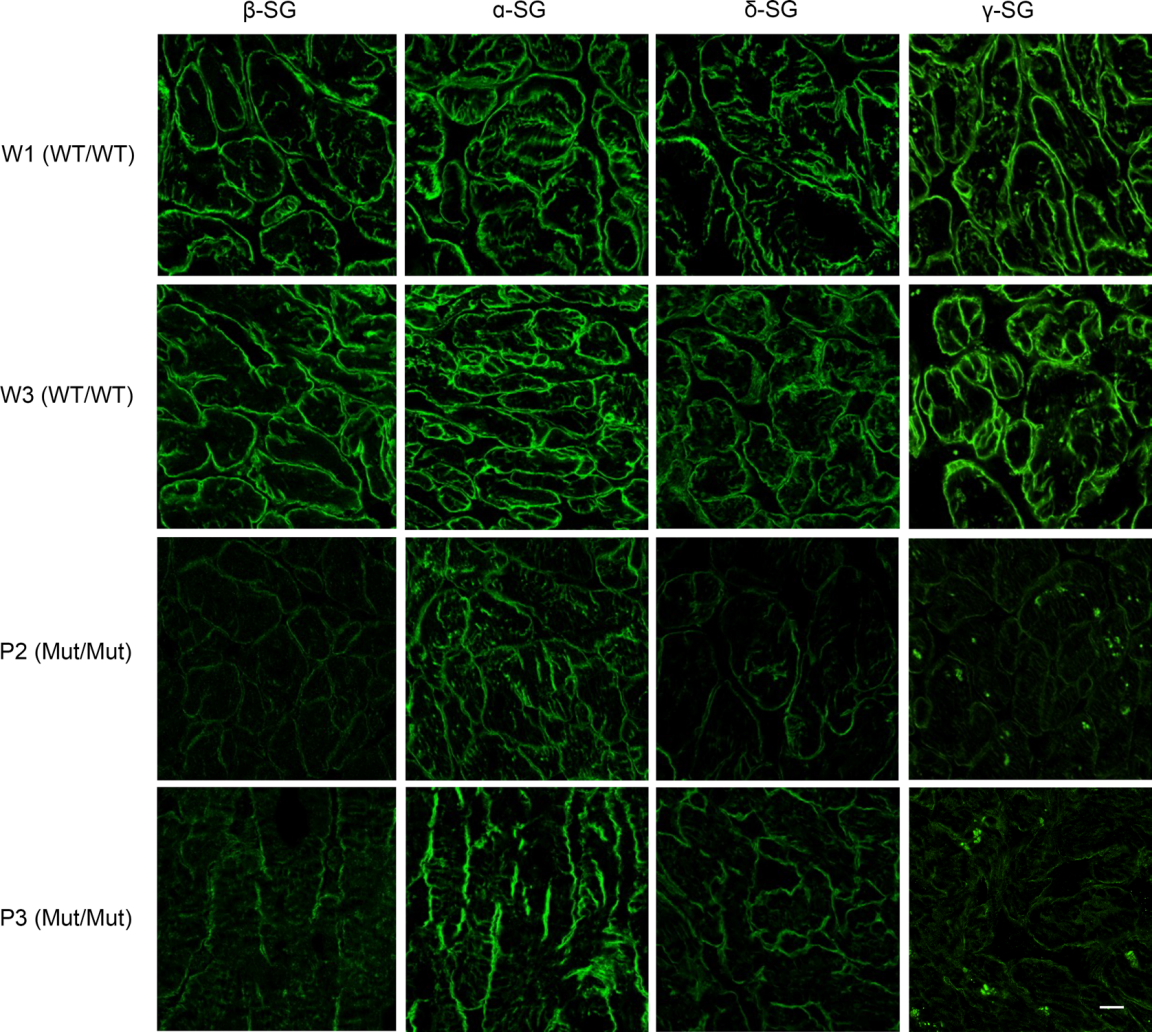
Immunohistochemical analysis of sarcoglycan subunits in cardiac tissue. Cardiac sections from samples carrying the WT (WT/WT) or homozygous c.243+6T>A (Mut/Mut) allele of *SGCB* were stained with antibodies against β-SG, α-SG, δ-SG, and γ-SG. Representative images from 2 WT/WT and 2 Mut/Mut samples are shown. Images were acquired at ×1,000 magnification. Scale bar: 20 μm.

Consistent with these results, immunohistochemical analysis revealed markedly reduced membrane localization of β-SG and other sarcoglycan subunits in Mut/Mut samples (Figure 4), although the reduction in α-SG signal was relatively mild in line with the Western blot analysis. Notably, despite the overall reduction, partial membrane localization of β-SG was retained, colocalizing with γ-SG, representative of other subunits (Supplemental Figure 9). Collectively, these results indicate that exon 2 skipping of *SGCB* markedly reduces membrane localization of β-SG and compromises the overall stability of the sarcoglycan complex.

### Muscle strength, serum creatine kinase levels, and skeletal muscle imaging reveal no evidence of myopathy in patients with the c.243+6T>A variant.

**Figure 5:**
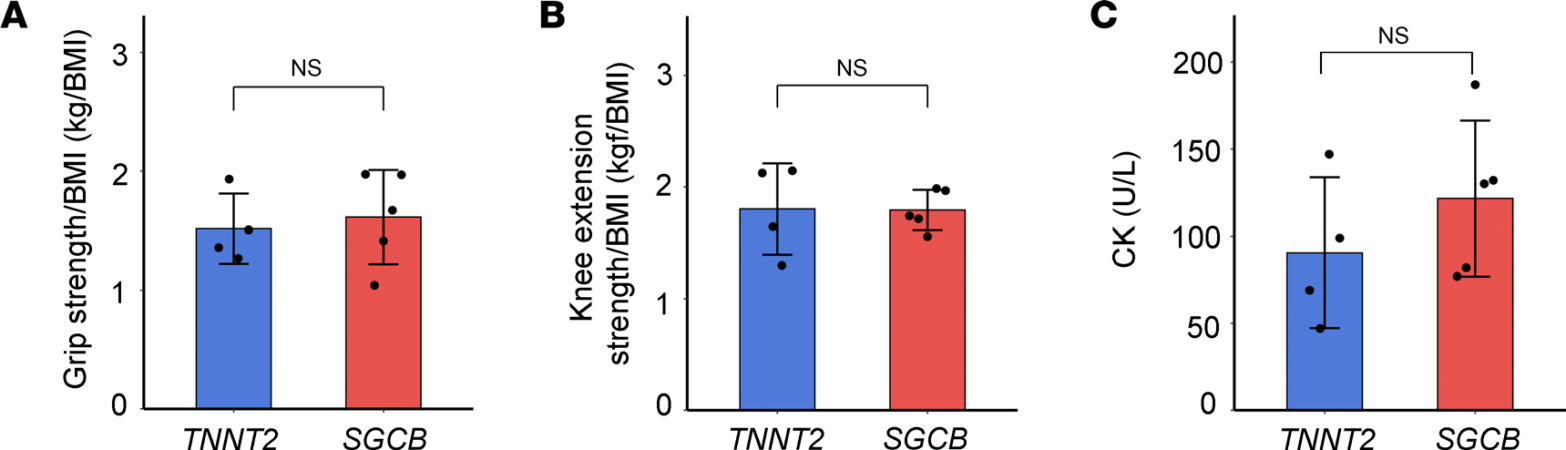
Muscle strength and serum CK levels in patients with *SGCB* and *TNNT2* variants. (**A**) Muscle strength assessed by grip strength/BMI and (**B**) knee extension strength/BMI showed no significant differences between the 2 groups. (**C**) Serum CK levels between the groups; the normal reference range is 54–286 U/L. *TNNT2* and *SGCB* refer to patients with the c.407G>A variant in *TNNT2* (*n* = 4) and the c.243+6T>A variant in *SGCB* (*n* = 5), respectively. Data are presented as the mean ± SD. *P* values were calculated using a 2-tailed Welch’s *t* test.

To our knowledge, all the variants of *SGCB* have been reported to be associated with clinical symptoms of LGMD, typically characterized by progressive muscle weakness — particularly in proximal muscle groups such as the thigh or upper arm — and marked elevation of creatine kinase (CK) levels (16). We thus compared muscle strength between patients homozygous for the *SGCB* c.243+6T>A variant and those heterozygous for the *TNNT2* c.407G>A variant (NM_001276345), which is theoretically not associated with muscle symptoms due to its cardiac-specific expression. The 2 groups showed no significant differences in age (*SGCB*, 42 ± 7.1 years vs. *TNNT2*, 45.3 ± 11.9 years, *P* = 0.65) or left ventricular ejection fraction (LVEF; *SGCB*, 21.4 ± 5.7% vs. *TNNT2*, 22.0 ± 3.4%, *P* = 0.85). Under these conditions, no significant differences in grip strength or knee extension strength, both adjusted for BMI, were observed between the groups (Figure 5, A and B, and Supplemental Table 2). In addition, serum CK levels were comparable between the groups (Figure 5C and Supplemental Table 2), and CK levels for all 12 patients carrying the c.243+6T>A variant were within the normal range (Supplemental Table 3).

To further evaluate skeletal muscle involvement at the tissue level, we performed native T1 mapping using cardiac MRI, a quantitative technique in which elevated T1 values reflect tissue pathology such as fibrosis or fatty infiltration (17). We analyzed T1 values of skeletal muscles (serratus anterior and pectoralis major) incidentally captured during routine cardiac MRI in patients with DCM carrying the homozygous *SGCB* c.243+6T>A variant and age- and sex-matched healthy male patients acting as controls. While myocardial native T1 values were significantly higher in DCM patients than in controls (*P* = 0.011), skeletal muscle T1 values showed no significant difference between the 2 groups for either the serratus anterior (*P* = 0.759) or the pectoralis major (*P* = 0.179) (Supplemental Figure 10 and Supplemental Table 4). The absence of muscle weakness and normal skeletal muscle T1 values in the *SGCB* group strongly supports the association of the homozygous c.243+6T>A variant with isolated DCM, without skeletal muscle involvement.

### Human-induced pluripotent stem cell–derived skeletal myocytes show reduced β-SG with relative preservation of the sarcoglycan complex.

**Figure 6:**
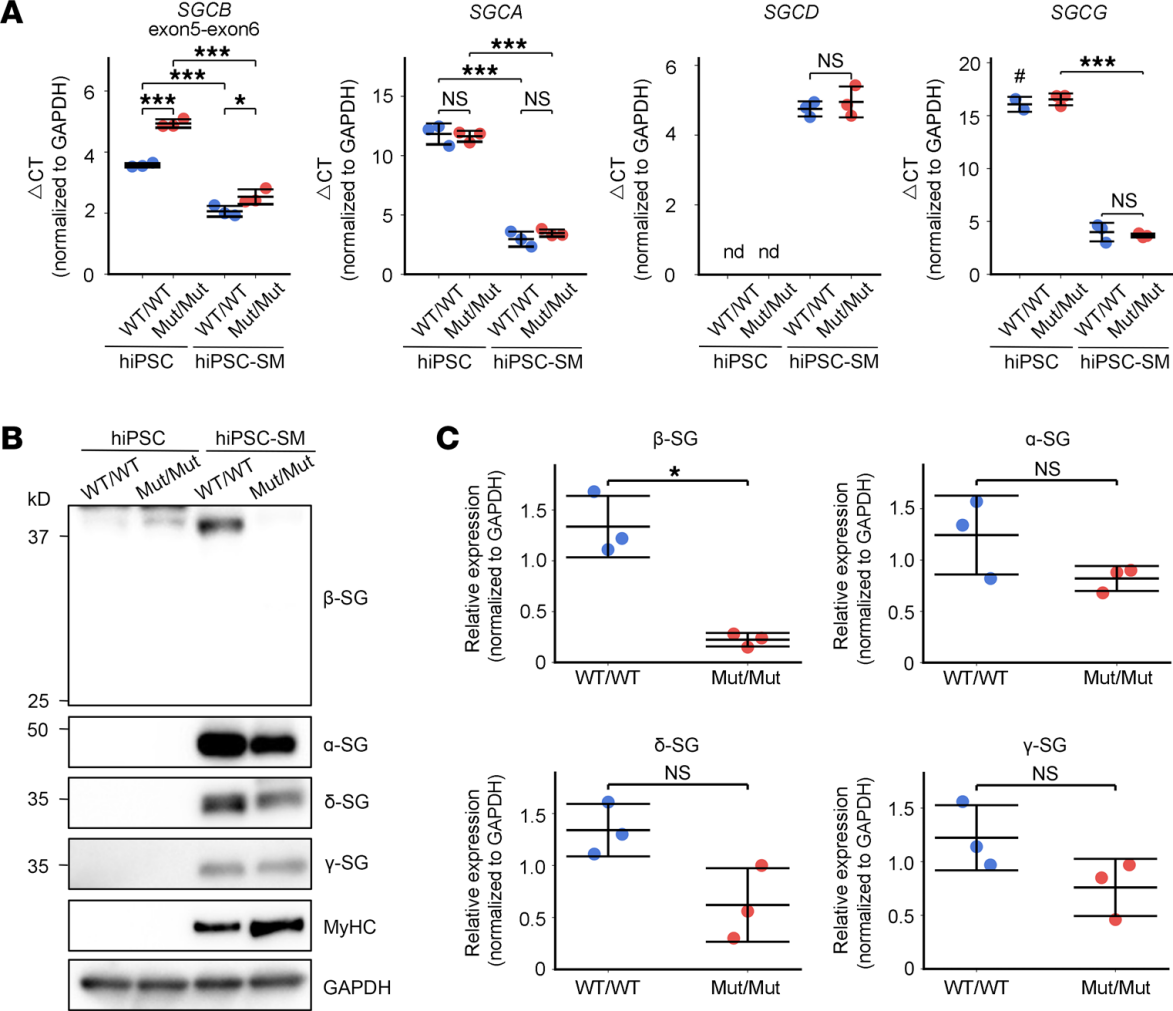
Sarcoglycan subunit expression in hiPSC–derived skeletal myocytes. (**A**) qPCR analysis of hiPSCs and hiPSC-derived skeletal myocytes (hiPSC-SMs) from a control line (253G1; WT/WT) and a patient carrying the homozygous *SGCB* c.243+6T>A variant (Mut/Mut) (*n* = 3 independent experiments). Expression levels are presented as ΔCt values normalized to GAPDH; lower ΔCt values indicate higher expression. #, Only 2 samples were analyzed because 1 sample was undetermined. (**B**) Representative Western blot analysis of β-SG, α-SG, δ-SG, and γ-SG in hiPSCs and hiPSC-SMs from WT/WT and Mut/Mut lines. Myosin heavy chain (MyHC) was used as a differentiation marker, and GAPDH was used as a loading control. (**C**) Quantification of Western blot band intensities of hiPSC-SMs shown in **B**, performed using ImageJ (*n* = 3 independent experiments). Data are presented as the mean ± SD. *P < 0.05; ***P < 0.001. *P* values were calculated using 1-way ANOVA followed by the Tukey–Kramer test for pairwise comparisons in **A** and using a 2-tailed Welch’s *t* test in **C**.

To investigate the impact of the c.243+6T>A variant on the sarcoglycan complex in skeletal muscle, we generated human-induced pluripotent stem cells (hiPSCs) from a homozygous patient (Mut/Mut) (Supplemental Figure 11) and differentiated them, along with a control line (253G1; WT/WT), into skeletal myocytes (Supplemental Figure 12). All 4 sarcoglycan genes were upregulated upon differentiation (Figure 6A). Among differentiated skeletal myocytes, expression levels of *SGCA*, *SGCD*, and *SGCG* were comparable between WT/WT and Mut/Mut, whereas *SGCB* expression measured with an exon 5–6 qPCR assay was slightly but significantly elevated in Mut/Mut cells (Figure 6A).

PCR analysis confirmed that *SGCB* exon 2 skipping occurred in Mut/Mut cells both in the undifferentiated hiPSC state and after skeletal myocyte differentiation, and exon 2–containing *SGCB* transcripts were markedly decreased in Mut/Mut compared with WT/WT (Supplemental Figure 13, A and B). Furthermore, RT-PCR spanning the full *SGCB* coding region detected no transcript isoforms beyond the exon 2–skipped product in Mut/Mut cells, indicating the absence of compensatory splicing events (Supplemental Figure 13C).

Western blot analysis revealed that β-SG protein was markedly reduced in Mut/Mut skeletal myocytes (Figure 6, B and C). In contrast, α-, γ-, and δ-SG showed a trend toward reduction but did not reach statistical significance (Figure 6, B and C). This pattern differed from that observed in patient cardiac tissue, where all sarcoglycan subunits were significantly reduced (Figure 3, B and C), suggesting that the downstream impact of reduced β-SG expression on the remaining complex components may be less severe in skeletal muscle.

### The homozygous SGCB c.243+6T>A variant is associated with poor prognosis in DCM.

Genetic background is known to influence clinical outcome in DCM (3, 18). To investigate the clinical impact of the *SGCB* c.243+6T>A variant, we compared the 12 patients carrying biallelic pathogenic *SGCB* variants (11 homozygous and 1 compound heterozygous) with 50 variant-negative (V–) DCM patients, defined as those lacking pathogenic variants in any of the 40 known DCM-associated genes (see Methods), and with 105 DCM patients carrying pathogenic *TTN* truncating variants, selected as the comparator group because they represent the most common genetic cause of DCM (19). The *SGCB* group tended to be diagnosed at a slightly younger age (41 years [IQR 32–48]) than the V– group (49 years [IQR 45–55]) and the *TTN* group (46 years [IQR 35–58]) (Table 1 and Supplemental Table 5). While no significant difference was observed in LVEF between the groups, the left ventricular end-diastolic diameter was significantly greater in the *SGCB* group (72 mm [IQR 66–80]) than in the V– group (65 mm [IQR 61–71], *P* = 0.007), and the *TTN* group (66 mm [IQR 62–72], *P* = 0.016) (Table 1 and Supplemental Table 5). In contrast, no significant differences were observed between the groups in sex distribution, family history of cardiomyopathy, or the use of major heart failure medications (Table 1).

**Figure 7:**
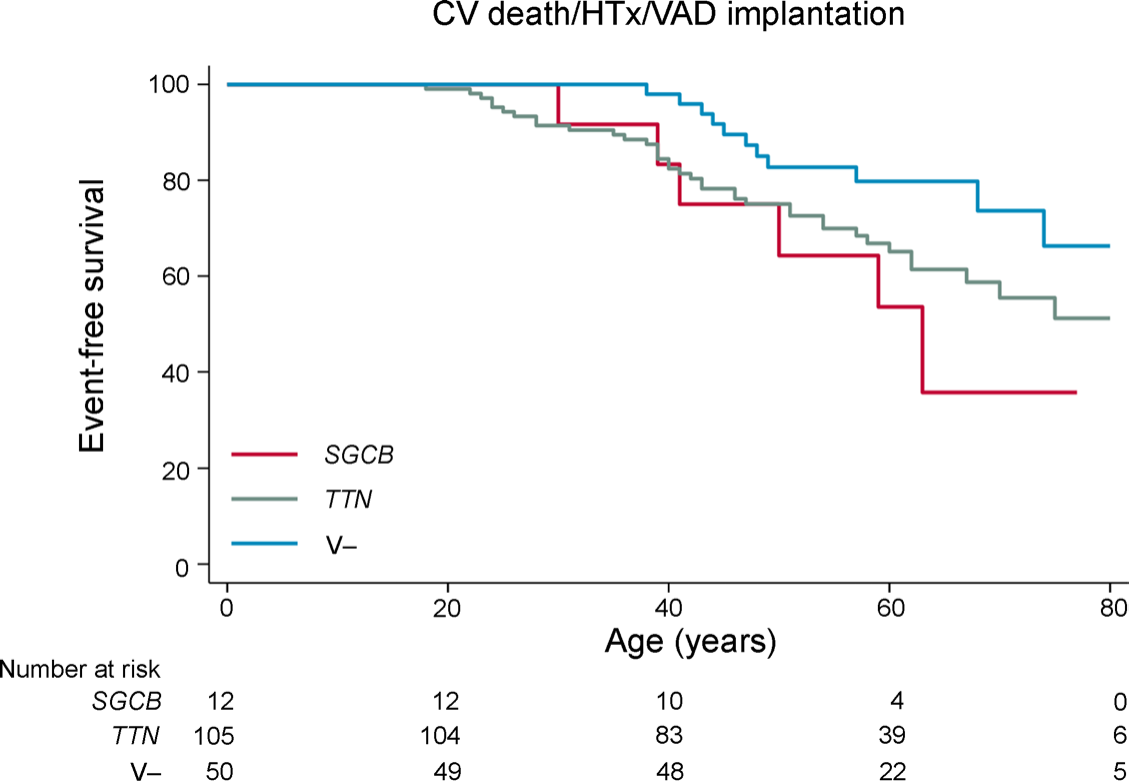
Event-free survival in patients with DCM harboring biallelic pathogenic *SGCB* variants, without known pathogenic variants or with *TTN* variants. Patients with DCM were divided into 3 groups: the *SGCB* group (*n* = 12), consisting of patients with biallelic pathogenic *SGCB* variants (11 homozygous for c.243+6T>A and 1 compound heterozygous); the variant-negative (V–) group (*n* = 50), consisting of patients without pathogenic variants in any of the 40 known DCM-associated genes; and the *TTN* group (*n* = 105), consisting of patients with *TTN* variants classified as P/LP according to ACMG criteria. Kaplan-Meier curves illustrate survival free of the composite endpoint — cardiovascular (CV) death, HTx, or VAD implantation — using age as the timescale. *P* values were calculated using a univariable Cox proportional hazard model. The number of patients at risk is shown below the Kaplan-Meier curves.

Kaplan-Meier analysis using age as the timescale revealed a significantly earlier onset of the composite endpoint — VAD implantation, HTx, or cardiovascular death — in the *SGCB* group compared with the V– group (Figure 7; HR, 2.90; 95% CI, 1.07–7.88; *P* = 0.036). The *SGCB* group also showed a trend toward earlier adverse events compared with the *TTN* group (HR, 1.47; 95% CI, 0.62–3.49; *P* = 0.383), although this comparison did not reach statistical significance, likely owing to the limited sample size of the *SGCB* group. These findings suggest that the presence of the *SGCB* variant is associated with an increased risk of early adverse outcomes.

Collectively, these findings indicate that the homozygous *SGCB* c.243+6T>A variant defines a high-risk subset of patients with DCM, characterized by an enlarged left ventricle and increased risk of adverse clinical outcomes.

## Discussion

Through integrated RNA-seq and WGS analyses, we demonstrated that the splice-site variant (c.243+6T>A) in *SGCB*, which induces exon 2 skipping, is a genetic cause of DCM. In cardiac tissue from patients homozygous for the c.243+6T>A variant, we observed markedly reduced expression and membrane localization of β-SG and other sarcoglycan complex components. Furthermore, homozygosity for this variant was significantly associated with greater left ventricular dilation and a higher risk of early-onset adverse cardiac events, including VAD implantation, HTx, and cardiovascular death. This suggests that c.243+6T>A defines a high-risk subset of DCM.

The pathogenicity of a genetic variant is typically evaluated based on multiple lines of evidence, including allele frequency, in silico prediction, variant pathogenicity databases, segregation data, and functional studies, in accordance with ACMG guidelines (14). The *SGCB* c.243+6T>A variant exhibited a high SpliceAI score of 0.78, indicating a strong splicing impact (20). Nevertheless, its relatively high allele frequency (0.9%) in the East Asian population of gnomAD and the presence of 3 homozygous individuals in a Japanese general population cohort (ToMMo) previously led to its classification as likely benign. However, our study demonstrated a significant enrichment of homozygous c.243+6T>A in the DCM cohort, consistent segregation of the genotype with the phenotype across multiple families (Figure 2), and clear protein-level evidence indicating a loss-of-function effect in cardiac tissue (Figures 3 and 4). These findings warrant a reevaluation of its pathogenicity and strongly support the conclusion that homozygosity for the c.243+6T>A variant plays a direct role in the pathogenesis of DCM.

Biallelic loss-of-function variants in *SGCB* have previously been associated with LGMDR4, one of the most severe LGMD subtypes (12). Most patients with LGMDR4 develop symptoms before the age of 10, with progressive muscle weakness that typically leads to loss of ambulation by late adolescence (16). Because the sarcoglycan complex is expressed in cardiac muscle as well as skeletal muscle, about 40% of LGMDR4 patients develop DCM, with a mean age of onset at 25.8 years. Furthermore, homozygous *SGCB*-knockout mice develop a combination of skeletal myopathy and cardiomyopathy (21, 22), supporting a mechanistic link between sarcoglycan complex disruption and both skeletal and cardiac dysfunction. However, in contrast to LGMDR4 patients and *SGCB*-knockout models, homozygous individuals for the c.243+6T>A variant in this study exhibited no clinical signs of skeletal muscle involvement (Figure 5). Objective muscle strength measurements and serum CK levels also revealed no abnormalities indicative of skeletal muscle involvement. These findings suggest that the phenotype associated with the c.243+6T>A variant is markedly distinct from that of LGMDR4 and is instead characterized by a cardiac-specific phenotype.

The mechanism by which individuals homozygous for the *SGCB* c.243+6T>A variant do not exhibit overt skeletal muscle symptoms despite developing DCM is not fully understood. It is well established that loss of *SGCB* expression in skeletal muscle consistently leads to severe myopathy (16). However, Western blot analysis of hiPSC-derived skeletal myocytes from a homozygous patient showed that, although β-SG protein was markedly reduced, α-, γ-, and δ-SG levels were not significantly decreased (Figure 6, B and C). This contrasts with patient cardiac tissue, where all sarcoglycan subunits were significantly reduced (Figure 3, B and C), suggesting that the sarcoglycan complex may be relatively preserved in skeletal muscle despite reduced β-SG. Exon 2 of *SGCB* encodes a portion of the intracellular and transmembrane domains of β-SG (23). Consistent with in silico topology prediction, exon 2 skipping is predicted to remove most of the transmembrane segment; however, because the deletion is in-frame, the extracellular domain remains largely intact. Although these structural features are predicted to disrupt membrane anchoring, they alone do not readily explain the absence of overt skeletal muscle involvement. One potential explanation for the tissue-specific phenotype is compensatory alternative splicing, as reported in dystrophinopathies, where intronic rearrangements can cause splicing errors preferentially in cardiac muscle (24). However, RT-PCR analysis spanning the full coding region in hiPSC-derived skeletal myocytes detected only the exon 2–skipped isoform, with no additional compensatory transcript isoforms (Supplemental Figure 13C). Although hiPSC-derived skeletal myocytes may not fully recapitulate the maturation state of adult skeletal muscle in vivo, these findings suggest that the cardiac-specific phenotype is more likely attributable to tissue-specific differences in protein stability or complex assembly — potentially reflecting differences in membrane composition, interacting partners, or mechanical load — rather than compensatory alternative splicing.

Notably, homozygous patients exhibited a marked reduction in β-, δ-, and γ-SG expression, whereas the decrease in α-SG expression was relatively modest (Figures 3 and 4). The sarcoglycan complex forms a stable trimeric core composed of β-, δ-, and γ-SG, with α-SG functioning as a peripheral component (25). While loss of any core component typically destabilizes the entire complex, selective reduction of α-SG expression with preservation of the other 3 subunits has also been reported (26, 27). These observations suggest that the relatively mild decrease in α-SG expression observed in this study may reflect a weaker interaction between β- and α-SG compared with the stronger interactions among β-, δ-, and γ-subunits.

Emerging therapeutic strategies for DCM, such as gene replacement using adeno-associated virus vectors and genome editing via CRISPR/Cas9, have demonstrated efficacy in animal models and are advancing through preclinical stages (3, 28). Considering the loss-of-function effect of c.243+6T>A in *SGCB*, these approaches represent promising therapeutic options. Additionally, splice-switching oligonucleotides (SSOs), which correct aberrant splicing, offer the advantage of modulating gene expression, without altering the genome or inducing target gene overexpression (29). Several SSOs have already been implemented in clinical practice, including Spinraza, which is approved for the treatment of spinal muscular atrophy (30). Although efficient delivery to cardiac tissue remains a challenge, SSOs still hold considerable potential as an effective therapeutic approach for correcting aberrant exon skipping in *SGCB*.

Analysis of the UK Biobank Allele Frequency Browser showed that the *SGCB* c.243+6T>A variant is highly enriched in East Asians (MAF ~0.94%), present at low frequency in South Asians, and absent in Europeans (MAF = 0), consistent with gnomAD v4.1.0. These findings suggest that this variant represents a predominantly East Asian–specific contributor to DCM. Given this relatively high allele frequency, homozygosity for the c.243+6T>A variant may underlie a non-negligible number of DCM cases in this ethnic background, underscoring the clinical importance of developing targeted therapies. Importantly, none of the heterozygous carriers in our study exhibited a DCM phenotype, implying that even partial correction — through gene editing, restoration of gene expression, or splicing correction — could offer therapeutic benefit. Our genetic enrichment analyses were performed using a single Japanese DCM cohort and a single population-based reference cohort, and independent replication in additional DCM cohorts — particularly those with sufficient representation of East Asian individuals — is needed to confirm the generalizability of this association. We hope that the findings of this study will contribute to the future development of such targeted therapies in *SGCB* c.243+6T>A-associated DCM.

## Methods

### Sex as a biological variable.

This study included both male and female patients. Sex was not a factor in participant selection, and the study was designed to be applicable to both sexes.

### Study participants for transcriptomic outlier analysis.

For the transcriptomic outlier analysis using RNA-seq data and the DROP workflow, 107 patients who underwent HTx or VAD implantation at Osaka University Hospital were included (female, *n* = 33; male, *n* = 74). Among them, 63 patients had been diagnosed with idiopathic DCM. To exclude transcriptomic changes commonly associated with end-stage heart failure and to enhance the statistical power of the analysis, 44 additional patients with nonidiopathic DCM were also included. These nonidiopathic DCM cases comprised dilated-phase hypertrophic cardiomyopathy (*n* = 10), ischemic cardiomyopathy (*n* = 10), drug-induced cardiomyopathy (*n* = 4), postmyocarditis DCM (*n* = 4), restrictive cardiomyopathy (*n* = 4), peripartum cardiomyopathy (*n* = 3), cardiac sarcoidosis (*n* = 2), Becker muscular dystrophy (*n* = 2), left ventricular noncompaction (*n* = 2), arrhythmogenic right ventricular cardiomyopathy (*n* = 1), fulminant myocarditis (*n* = 1), and double outlet right ventricle (*n* = 1).

DROP identifies aberrant expression and splicing events by comparing each sample to the distribution of all other samples within the cohort; thus, the fold change threshold (≤0.5) reflects deviation from the cohort-wide distribution rather than comparison with a predefined control group. The complete DROP output for all 107 samples is provided in the Supporting Data Values file (tabs Fig.1A-1 and Fig.1B-1). Idiopathic DCM was defined as the presence of left ventricular systolic dysfunction (LVEF < 50%) and left ventricular dilation, after thorough exclusion of secondary causes based on comprehensive clinical evaluation, as previously described (1, 31).

### Prioritization of candidate genes in genetically undiagnosed DCM.

Idiopathic DCM cases were first screened for pathogenic variants within a curated panel of 40 DCM-associated genes using WES. This panel included 36 genes from 2 recent landmark studies on DCM (4, 31), chosen as an evidence-based reference set for DCM, and was supplemented with 4 additional genes (*BAG5*, *LMOD2*, *PPCS*, and *RRAGD*) selected from the GenCC database based on a prespecified criterion of DCM gene–disease validity rated as “moderate” or higher by at least 2 independent submitter organizations (https://thegencc.org/). Cases harboring variants classified as P/LP according to the ACMG criteria were considered genetically diagnosed.

To identify candidate genes in the remaining genetically undiagnosed cases, transcriptomic outlier analysis was applied to RNA-seq data from the 107 patients described above using the DROP workflow, as described in *RNA-seq and analysis*. RNA-seq and WES were performed uniformly across the entire cohort (*n* = 107), while WGS was selectively applied to samples identified as outliers.

Among the aberrant splicing and expression outliers detected by the DROP workflow, candidate gene prioritization was performed separately for each outlier type. For splicing outliers, WGS data were analyzed to identify genes harboring rare variants (MAF < 0.01 for homozygous and < 0.001 for heterozygous variants) located within aberrantly spliced regions and their flanking ±100 bp. Among these, genes carrying variants with a high predicted splicing impact, as indicated by a SpliceAI score ≥ 0.5 (20), and sufficient cardiac expression (normalized AUC: NAUC > 1, an objective filter to exclude genes with negligible myocardial expression), a metric derived from ASCOT (32, 33), were prioritized as final candidates (Supplemental Figure 1). For expression outliers (fold change ≤ 0.5), WGS data were analyzed to identify genes harboring rare variants (MAF < 0.01 for homozygous and < 0.001 for heterozygous variants) predicted to affect gene expression, such as frameshift, splice site, nonsense mutations, or large deletions. Among these, genes with sufficient cardiac expression (NAUC > 1) were prioritized as final candidates (Supplemental Figure 1). MAF values were assessed using the highest reported allele frequencies from population databases, including gnomAD (34), HGVD (35), and ToMMo (36).

### WES and WGS.

Genomic DNA was extracted from peripheral blood leukocytes. For WES, DNA was enzymatically fragmented using the Twist EF 2.0 Fragmentation Kit (Twist Bioscience), and libraries were prepared using the Twist Human Core Exome 2.0 and Mitochondrial Panel. Paired-end sequencing (2 × 150 bp) was performed on a NovaSeq X system (Illumina). For WGS, DNA libraries were prepared using the TruSeq DNA PCR-Free Library Prep Kit (Illumina), and sequencing was performed on a NovaSeq 6000 system (Illumina). For both WES and WGS, adapter sequences and low-quality bases were trimmed using fastp (v0.23.4). The processed reads were aligned to the human reference genome (GRCh38) using Burrows-Wheeler Aligner (v0.7.17). Variant calling was performed according to the GATK Best Practices workflow (v4.2.0.0), including duplicate marking (MarkDuplicates), base quality score recalibration, and variant detection with HaplotypeCaller. Variants were annotated using ANNOVAR (version dated June 2020; http://annovar.openbioinformatics.org/). CNVs were evaluated in patients who underwent WGS. CNVs were detected using CNVpytor (v1.3.1), Manta (v1.6.0), Delly (v1.2.6), and the smoove pipeline (v0.2.8). CNV calls from individual tools were merged on a per-sample basis and annotated using AnnotSV (v3.5.3). Variant pathogenicity was assessed according to the ACMG/ClinGen guidelines (37). Deletions overlapping exons of genes associated with DCM and classified as pathogenicity class ≥ 3 were extracted as candidate disease-causing variants. Candidate CNVs were subsequently inspected using Integrative Genomics Viewer to exclude potential false positives and to confirm CNV boundaries.

### RNA-seq and analysis.

Total RNA was extracted from patient-derived cardiac tissue using the miRNeasy Mini Kit (Qiagen, catalog 1038703). RNA libraries were prepared using the TruSeq stranded mRNA sample prep kit (Illumina) according to the manufacturer’s instructions. Whole-transcriptome sequencing was performed on the HiSeq 3000 platform using the 100 bp paired-end mode. Sequence reads were aligned to GRCh38 reference with STAR v2.7.1. Read group tags were added, and BAM index files (BAI) were generated using Samtools (v1.10). Raw read counts were quantified using Stringtie (v2.1.1). Quality control was performed using RNA-SeQC (v5.0.1) and multiqc (v1.14), and only samples with transcript integrity number values > 60 were retained (38).

Analysis of RNA-seq data was performed using DROP v1.2.4 (10), which integrates OUTRIDER and FRASER for systematic detection of transcriptomic aberrations. Aberrant gene expression was detected using OUTRIDER (v1.16.1, padjCutoff = 0.05, zScoreCutoff = 2) across the 107 samples described above (39), and aberrant splicing was detected using FRASER (v1.8.1, padjCutoff = 0.1, zScoreCutoff = 2, deltaPsiCutoff = 0.3) with the same samples (40). Sample identity between RNA and DNA was confirmed using the sample matching functionality implemented in the DROP pipeline. Exon usage analysis was additionally performed using DEXSeq (v1.44.0), a tool for detecting differential exon usage from RNA-seq data (13).

### PCA-based population structure analysis.

To evaluate potential confounding by population stratification between the DCM cohort and the population-based reference cohort, we assessed genome-wide genetic ancestry using PCA. We compared the NCVC-GSN DCM cohort (*n* = 936) with the ToMMo-54KJPN reference population (*n* = 54,212). Related individuals up to the second degree were excluded prior to analysis. Given the available shared variant set, we performed a projection PCA framework. Briefly, PCA was performed in the ToMMo-54KJPN reference population using a predefined list of 21,925 shared SNPs, and the NCVC-GSN samples were then projected onto the same principal component axes using PLINK2. Principal components (PC1–PC10) derived from this framework were used as ancestry covariates in downstream association analyses.

### RT-PCR, qPCR, and dPCR analysis.

Total RNA was extracted from cardiac tissue using the miRNeasy Mini Kit and converted to cDNA using the PrimeScript RT Reagent Kit (TaKaRa, catalog RR037A) according to the manufacturer’s protocols. The target DNA segment was amplified by PCR using TaKaRa Ex premier DNA Polymerase (RR370S). Primer sequences are provided in Supplemental Table 6. PCR products were resolved on a 2% agarose gel (Invitrogen, catalog 16500500) in TBE buffer (0.09 M Tris, 0.09 M boric acid, and 0.002 M EDTA, pH 8.0), stained with GelRed nucleic acid stain (Biotium), and visualized under UV illumination. To verify the identity of each PCR product, the target bands were excised from the gel, DNA was purified, and Sanger sequencing was performed.

qPCR was performed using TaqMan probe specific for *SGCB* (exon 2-3 and exon 5-6), *SGCA*, *SGCD*, *SGCG*, and GAPDH. A 96 well plate (BIO-BIK) was used, and reactions were performed by using TaqMan Fast Advanced Master Mix for qPCR (Thermo Fisher Scientific, catalog 4444557).

dPCR was performed using a TaqMan probe specific for *SGCB* (Thermo Fisher Scientific, Hs01086035_m1). Reactions were conducted on the Absolute Q Universal DNA Digital PCR System (Thermo Fisher Scientific) with the Absolute Q Universal DNA Digital PCR Master Mix 5X (catalog A72710), using the following thermal cycling conditions: initial denaturation at 96°C for 10 min, followed by 40 cycles of 96°C for 5 s and 60°C for 20 s.

### Genetic screening for SGCB c.243+6T>A variants in a Japanese DCM cohort.

WES or WGS data from 921 patients with DCM who had undergone genetic testing at Osaka University Hospital (*n* = 412) or the National Cerebral and Cardiovascular Center (*n* = 509) were analyzed to identify individuals harboring heterozygous or homozygous *SGCB* c.243+6T>A variants. All 12 patients identified as homozygous for the *SGCB* variant were confirmed by Sanger sequencing, and their clinical information is summarized in Supplemental Table 5.

### Familial segregation analysis of SGCB variants.

For familial analysis, DNA from the proband and family members was extracted from peripheral blood leukocytes or saliva samples. Saliva-derived DNA was extracted using the ORA-gene-DISCOVER kit (DNAgenotek, OGR-675), according to the manufacturer’s instruction. Target regions were amplified by PCR using the primers listed in Supplemental Table 6. Sanger sequencing was subsequently performed to assess segregation of the *SGCB* variants within the family.

### Protein extraction and Western blotting.

For Western blotting, frozen heart tissue samples were ground into a fine powder using a Multi-Beads Shocker (Yasui Kikai) and lysed in SDS-PAGE sample buffer (50 mM Tris-HCl, pH 6.8, 20% SDS, and 9 M glycerol). The lysates were centrifuged at 20,000*g* for 10 min and rotated at 4°C for 15 min. HEK293T cells were washed with PBS and lysed directly in SDS-PAGE sample buffer. After centrifugation under the same conditions, the supernatants from both heart tissue and HEK293T cells were collected as soluble protein fractions. The supernatants were supplemented with 6% 2-mercaptoethanol and 0.002% bromophenol blue, sonicated using a Bioruptor UCW-310-EX (Cosmo Bio), and boiled at 70°C for 10 min, prior to analysis.

The protein samples were subjected to 12% SDS-PAGE and transferred onto PVDF membranes. After blocking with 3% nonfat milk for 30 min at room temperature, the membrane was incubated with solution containing primary antibody at 4°C overnight. The membranes were then washed with Tris-buffered saline with Tween 20 (TBS-T), incubated with corresponding anti-rabbit or anti-mouse secondary antibody at room temperature for 40 min, and washed again with TBS-T. Immunoreactive signals were detected using ECL Western Blotting Detection Reagents (GE Healthcare). Signal bands were then quantified using ImageJ (NIH, v1.54g). Primary antibodies used are as follows: β-SG (1:2,000, Fine Test, FNab00878), α-SG (1:1,000, Abcam, ab189254), γ-SG (1:1,000, Leica, NCL-g-SARC), δ-SG (1:1,000, Abcam, ab137101), GAPDH (1:3,000, Abcam, ab9485), and α–actinin 2 (1:2,000, Sigma-Aldrich, EA-53). For Ponceau S staining, transferred PVDF membranes were incubated in Ponceau S staining solution (Sigma-Aldrich, P7170) for 5 min to visualize protein transfer. After capturing an image of the stained membranes, they were rinsed with distilled water and briefly immersed in 0.1 M NaOH solution for 10–30 s.

### Cell culture and transfection.

HEK293T cells were purchased from American Type Culture Collection (ATCC CRL-11268) and prepared and cultured in DMEM with high glucose (Sigma-Aldrich), supplemented with 10% FBS and 1% penicillin-streptomycin. Cells were transfected with Lipofectamine 2000 (Invitrogen) and Opti-MEM (Gibco) according to the manufacturers’ protocols. Expression plasmids encoding untagged WT *SGCA* (NM_000023.4), *SGCD* (NM_000337.6), and *SGCG* (NM_000231.3) were constructed by VectorBuilder using a CMV promoter–driven expression vector.

### Generation of hiPSCs and differentiation into skeletal myocytes.

*SGCB* patient-derived hiPSCs were generated from PBMCs. Briefly, PBMCs were isolated from peripheral whole blood using Ficoll-Paque (GE Healthcare). Reprogramming was performed using Sendai virus vectors with OCT3/4, SOX2, KLF4, and c-MYC (CytoTune-iPS 2.0 Sendai Reprogramming Kit, Thermo Fisher Scientific). 24 h after transduction, PBMCs were seeded on a laminin-coated plate (iMatrix-511, Matrixome). hiPSCs were maintained on the laminin-coated plate with medium (StemFit AK02, Ajinomoto) (41). A control hiPSC line (253G1) was obtained from the RIKEN BioResource Research Center (Tsukuba, Japan).

Skeletal muscle cell differentiation of Tet-MyoD hiPSCs was performed according to the previously described protocol (42). Brieﬂy, 4 × 10^–5^ cells were seeded on Matrigel-coated (BD Biosciences, catalog 356231) 6 well plates (1:100) in StemFit medium (Takara Bio, StemFit AK02N) supplemented with 10 μM Y-27632. At 24 h after seeding, the medium was changed to primate embryonic stem cell medium (ReproCELL, RCHEMD001) without Y-27632. After 24 h, 0.3 μg/mL doxycycline (LKT Laboratories) was added to the culture medium. After an additional 24 h, the medium was changed into differentiation medium composed of α-MEM (with l-Gln, ribonucleosides, and deoxyribonucleosides, Nacalai Tesque, 21444-05) with 5% KSR (Invitrogen, 10828028), 0.5% Penicillin Streptomycin Mixed solution (Nacalai Tesque, 09367-34), 200 μM 2-mercaptoethanol, and 0.3 μg/mL doxycycline. Cells were cultured until day 7 with daily medium changes for Western blot and immunohistochemistry.

### Construction of SGCB expression plasmid.

The coding sequence of *SGCB* WT was amplified by PCR using cDNA from human heart tissue. The PCR product was inserted into the pCR II-Blunt-TOPO vector using the Zero Blunt TOPO PCR Cloning Kit (Thermo Fisher Scientific, 45-0245), and FLAG and HA tags were subsequently inserted at the N- and C-termini, respectively, using the NEBuilder HiFi DNA Assembly Cloning Kit (New England Biolabs, E5520S). The exon 2–skipped isoform of *SGCB* was generated from the *SGCB* WT vector by inverse PCR. Expression plasmids encoding FLAG- and HA-tagged *SGCB* WT and exon 2–skipped isoforms were constructed using the pSDH-SMV-MCS-EF1-puro vector (System Biosciences) according to the manufacturer’s protocol.

### Immunohistochemistry.

Left ventricular tissues were collected from patients with DCM during VAD implantation or HTx performed at Osaka University Hospital. A piece of left ventricular tissue was dissected and snap-frozen in liquid nitrogen. Sections from the heart tissue were then fixed with acetone for 20 min at 4°C. Next, the tissues were permeabilized with 0.1% Triton X-100 in PBS for 5 min and blocked with Blocking One Histo (Nacalai Tesque) for 10 min at room temperature. Then, the tissues were incubated overnight with primary antibodies at 4°C, followed by Alexa Fluor 488– or 594–labeled secondary antibodies (Invitrogen) for 45 min. Fluorescence images were recorded using a FluoView FV3000 confocal laser scanning microscope (Olympus). Primary antibodies used are as follows: β-SG (1:5,000, Fine Test, FNab00878), α-SG (1:200, Abcam, ab189254), γ-SG (1:50, Leica, NCL-g-SARC), and δ-SG (1:100, Abcam, ab137101).

For immunofluorescence staining of hiPSC-derived skeletal myocytes, cells were washed with PBS and permeabilized with 0.1% Triton X-100 in PBS for 5 min and blocked with Blocking One Histo for 10 min at room temperature. Cells were incubated overnight with primary antibodies at 4°C, followed by Alexa Fluor 488– or 594–labeled secondary antibodies (Invitrogen) for 45 min. Fluorescence images were acquired using a BZ-X810 microscope (Keyence). The anti–myosin heavy chain (MF20) primary antibody (1:100, Developmental Studies Hybridoma Bank) was used.

### Prediction of protein structure.

The transmembrane topology of the WT and exon 2–skipping *SGCB* proteins was predicted using TMHMM (v2.0). Three-dimensional structural models of the WT and exon 2–skipping *SGCB* proteins were generated using AlphaFold 3.

### Muscle strength assessment.

Muscle strength was evaluated in patients with the homozygous *SGCB* c.243+6T>A variant and compared with that of patients with a variant in the TNNT2 gene (c.407G>A, NM_001276345), which is theoretically not associated with muscle symptoms due to its cardiac-specific expression. Clinical parameters such as LVEF and serum CK levels shown in Figure 5 and Supplemental Table 2 were extracted from the time point closest to the muscle strength assessment. Skeletal muscle involvement was defined based on both CK measurement and documented muscle strength evaluation.

Grip strength (kg) was measured using a digital dynamometer (Takei Scientific Instruments, model T.K.K5401). Prior to measurement, the grip span was adjusted according to the patient’s hand size so that the second joint of the index finger formed a 90° angle with the handle. Patients were instructed to squeeze the handle with maximum effort using 1 hand. Measurements were performed twice for each hand alternately, and the final handgrip strength was defined as the average of the highest values recorded from the left and right hands, as previously described (43).

Knee extension strength (kfg) was measured using a handheld dynamometer (HHD; Anima Corp.). The patients were seated in a chair, keeping their trunks straight and vertical, with their hands resting on a bench beside their bodies. The HHD sensor was secured to the anterior side of the lower leg, between the level of the lateral ankle of the lower leg and the main post behind the chair. Isometric knee muscle extension force was measured for 5 s, at a 90-flexion angle. Measurements were repeated twice with an interval of at least 30 s, and the larger value was adopted. Each patient underwent testing 2–3 times, following a previously reported protocol (44). Grip strength and knee extension strength values were adjusted for individual differences using BMI.

### MRI analysis of myocardial and skeletal muscle native T1.

Cardiac magnetic resonance imaging was performed using a 1.5T Philips Ingenia scanner (Philips Healthcare) at Osaka University Hospital in 3 patients with DCM carrying an *SGCB* variant (*SGCB* group) and in 6 healthy subjects treated as controls. Control subjects were male and were selected to be as closely age-matched to the *SGCB* group as possible. Native T1 mapping was conducted according to standard institutional protocols. Within the *SGCB* group, 1 patient had previously undergone HTx; myocardial T1 was excluded, whereas skeletal muscle T1 was retained.

For myocardial T1, endocardial and epicardial contours of the left ventricle were traced, and the software automatically performed segmentation based on the right ventricular insertion points according to the American Heart Association 17-segment model to calculate the global mean T1 value. For skeletal muscle, regions of interest (ROIs) were placed on the serratus anterior (2–3 ROIs per subject; short-axis view) and the pectoralis major (1 ROI per subject; short-axis view). ROIs were manually drawn to exclude visible fat infiltration, fascial planes, and vascular structures.

### Clinical data collection and survival analysis.

Fifty individuals who lacked P/LP variants in any of the 40 known DCM-associated genes were randomly selected from the previously described DCM cohort. This was based on ACMG criteria, and they were designated the V– group. 105 DCM patients with variant of *TTN* were selected from the previously described DCM cohort. For all the 3 groups — *SGCB*, V–, and *TTN* — baseline clinical data including age, sex, family history of cardiomyopathy, New York Heart Association (NYHA) class, echocardiographic parameters, and medication use were collected from the time point closest to the age at diagnosis, as summarized in Table 1. The composite endpoint for Kaplan-Meier analysis was defined as cardiovascular death, HTx, or VAD implantation. Follow-up was censored at the earliest occurrence of a primary event or the last clinical contact.

### Statistics.

A summary of gene variants and clinical diagnoses for patients included in this study is provided in Supplemental Table 7.

Variables for baseline characteristics presented in Table 1 are expressed as median (interquartile range) or counts (%). Continuous variables were analyzed using the Mann-Whitney *U* test, and categorical variables were analyzed using Fisher’s exact test. Time-to-event outcomes were analyzed using univariable Cox proportional hazard models with age as the timescale, and Kaplan-Meier curves were generated for visualization.

For other quantitative analyses, data are presented as the mean ± SD. For 2-group comparisons, a 2-tailed Welch’s *t* test was used. Multiple group comparisons were performed using 1-way ANOVA, followed by Tukey-Kramer test for pairwise comparisons. Differences in carrier frequency between the ToMMo and DCM cohorts were evaluated using logistic regression adjusting for ancestry principal components (PC1–PC10). A *P* value < 0.05 was considered statistically significant.

### Study approval.

This study was performed in accordance with the ethical code approved by the Ministry of Health, Labor, and Welfare of Japan, and written informed consent was obtained from all participants or their guardians before inclusion in the study. The genome research protocol was approved by the Human Genome Research Bioethical Committee at The University of Osaka and the National Cerebral and Cardiovascular Center.

### Data availability.

All the data were derived from an in-house database of Osaka University Hospital and the National Cerebral and Cardiovascular Center. Values for all data points, excluding individual-level clinical data, are reported in the Supporting Data Values file. The WES, WGS, and RNA-seq datasets have not been deposited in a public database because of privacy and ethical limitations, and individual-level clinical data underlying the Kaplan-Meier analyses cannot be publicly shared because, even in deidentified form, they may carry a risk of reidentification and are restricted by institutional ethics approval and applicable privacy regulations. Access to these restricted datasets may be granted upon reasonable request to the corresponding author, subject to institutional review board approval.

## Author contributions

FL and H Shinomiya designed experiments. FL, H Shinomiya, and YK conducted experiments. FL, H Shinomiya, K Kanaoka, YI, Y Sakahashi, and TO collected and analyzed data. SI performed skeletal muscle MRI analysis. YYK, S Tadaka, INM, K Kinoshita, and KO contributed to ToMMo-54KJPN dataset analysis. H Sakurai contributed to hiPSC-based experimental work. YK, H Kioka, YM, KJ, H Kato, KM, KT, SN, S Takashima, YA, and Y Sakata advised on experimental design and data analysis. FL, H Shinomiya, and YA wrote the manuscript, integrating feedback from all coauthors.

## Conflict of interest

The authors have declared that no conflict of interest exists.

## Funding support

- Japan Agency for Medical Research and Development (AMED; JP25ek0109617, JP25ek0109639, JP25ak0101202, and JP25ek0109793 to YA and JP25ek0109760 to YA and Y Sakata).
- Grants-in-Aid from the Japan Society for the Promotion of Science (JSPS KAKENHI; JP23K24328 and JP25K02658 to YA and JP24K19057 to H Shinomiya).
- Japan Health Research Promotion Bureau (2024-B-08 to YA).
- Tohoku Medical Megabank Project is supported, in part, by the Ministry of Education, Culture, Sports, Science and Technology and Japan AMED (JP21km0105001, JP21km0105002, and JP21tm0424601).

## Supplementary Material

Supplemental data

Unedited blot and gel images

Supporting data values

## Figures and Tables

**Table 1:**
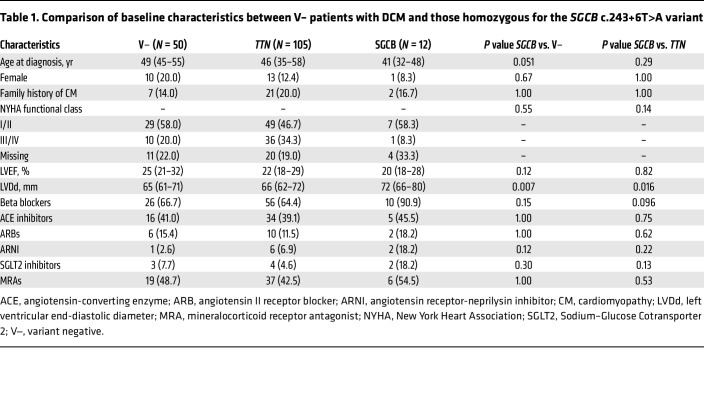
Comparison of baseline characteristics between V– patients with DCM and those homozygous for the *SGCB* c.243+6T>A variant

## Version 1 06/01/2026

Electronic publication
