# Supplementary material for: Homozygous SGCB splice-site variant causes isolated dilated cardiomyopathy through sarcoglycan complex destabilization in East Asians
Source: J Clin Invest. 2026 Jun 1;136(11):e198675. doi: 10.1172/JCI198675 (PMC13221219; doi:10.1172/JCI198675)
Supplement: Supplemental data [file jci-136-198675-s264.pdf]

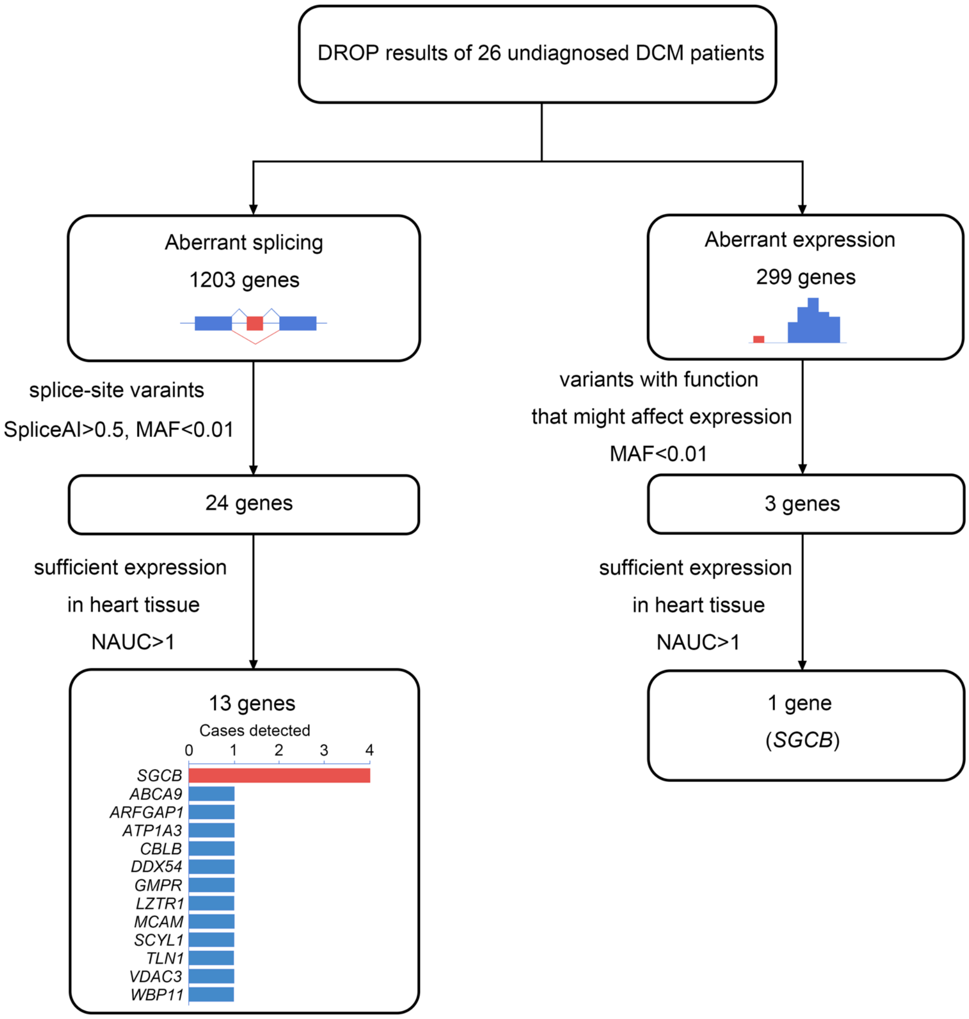

Supplemental Figure 1. Schematic workflow for narrowing down candidate causative genes for DCM. The bar graph indicates the number of cases with splicing abnormalities identified through the filtering process. DCM, Dilated cardiomyopathy; DROP, Detection of RNA Outliers Pipeline; MAF, Max Allele Frequency; NAUC, Normalized Area Under the Curve.

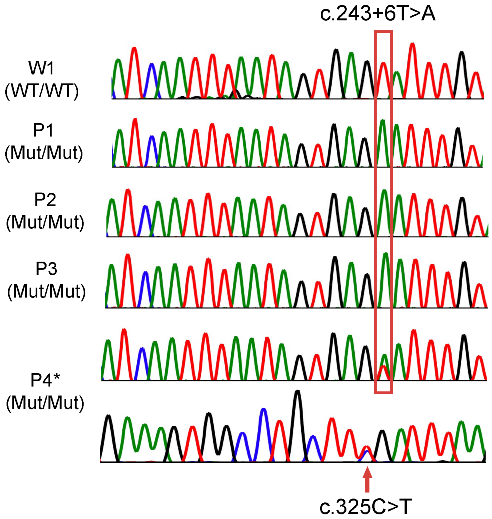

Supplemental Figure 2. Sanger sequencing chromatograms of patients harboring homozygous or two heterozygous *SGCB* variants identified by DROP (Detection of RNA Outliers Pipeline) analysis. W1 denotes a patient homozygous for the reference allele of *SGCB* at c.243+6T (WT/WT). P1–P3 are homozygous for the c.243+6T>A variant (Mut/Mut). P4* denotes a patient harboring two heterozygous variants: c.243+6T>A and c.325C>T (p.Arg109*).

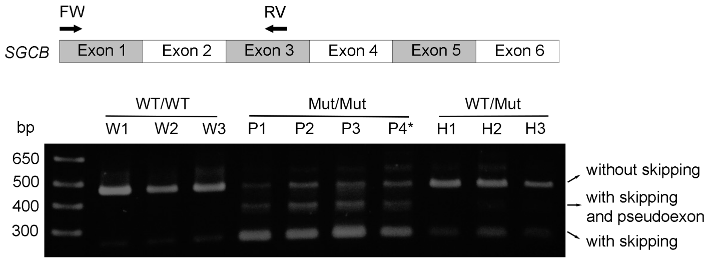

Supplemental Figure 3 PCR analysis of cDNA from cardiac tissue samples carrying the wild-type (WT/WT, n = 3), homozygous c.243+6T>A (Mut/Mut, n = 4), or heterozygous c.243+6T>A (WT/Mut, n = 3) allele of *SGCB*, using primers spanning Exon 1 to Exon 3. Three distinct PCR products were detected: the largest without Exon 2 skipping, the intermediate with Exon 2 skipping and pseudoexon inclusion, and the smallest with Exon 2 skipping alone.

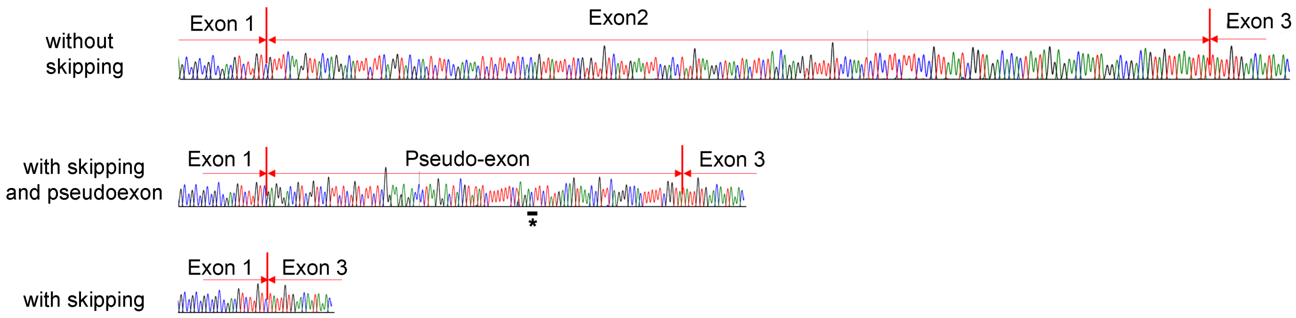

Supplemental Figure 4 Sanger sequencing chromatograms of three distinct PCR products of cDNA from a cardiac tissue sample homozygous for the c.243+6T>A variant (Mut/Mut), corresponding to Figure S3. For the isoform with skipping and pseudoexon, the predicted premature stop codon is indicated by an asterisk (*).

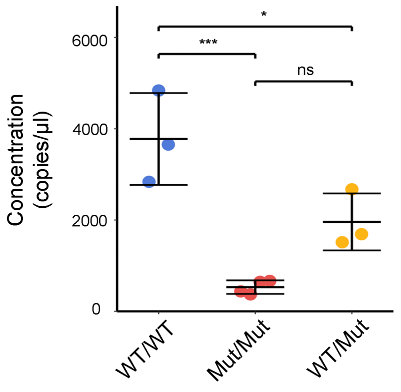

Supplemental Figure 5. Digital PCR (dPCR) analysis of *SGCB* transcripts containing exon 2 using cDNA extracted from cardiac tissue samples carrying the wild-type (WT/WT, n = 3), homozygous c.243+6T>A (Mut/Mut, n = 4), or heterozygous c.243+6T>A (WT/Mut, n = 3) allele. dPCR was performed using a TaqMan probe spanning the exon 2–exon 3 junction of *SGCB*, allowing specific detection of transcripts that include exon 2. Each dot represents the absolute concentration of exon 2–inclusive transcripts (copies/µl). Data are presented as mean ± SD. *$P < 0.05$; ***$P < 0.001$; ns, not significant.

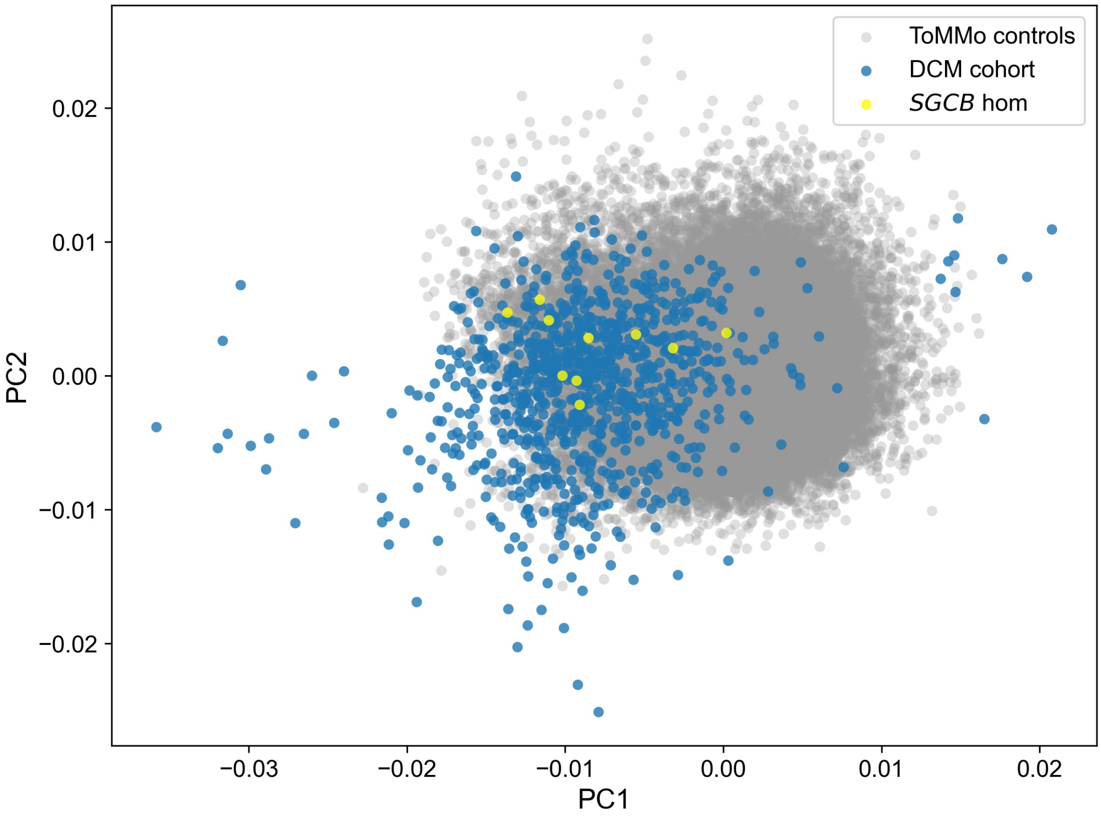

Supplemental Figure 6. Genome-wide principal component analysis (PCA) of the dilated cardiomyopathy (DCM) cohort and the Tohoku Medical Megabank Organization (ToMMo)-54KJPN reference population. PCA was computed using 21,925 shared SNPs. Principal component axes were derived in ToMMo-54KJPN (n=54,212), and DCM samples (n=936) were projected onto the same axes. DCM cases homozygous for the *SGCB* c.243+6T>A variant (n=10) are highlighted.

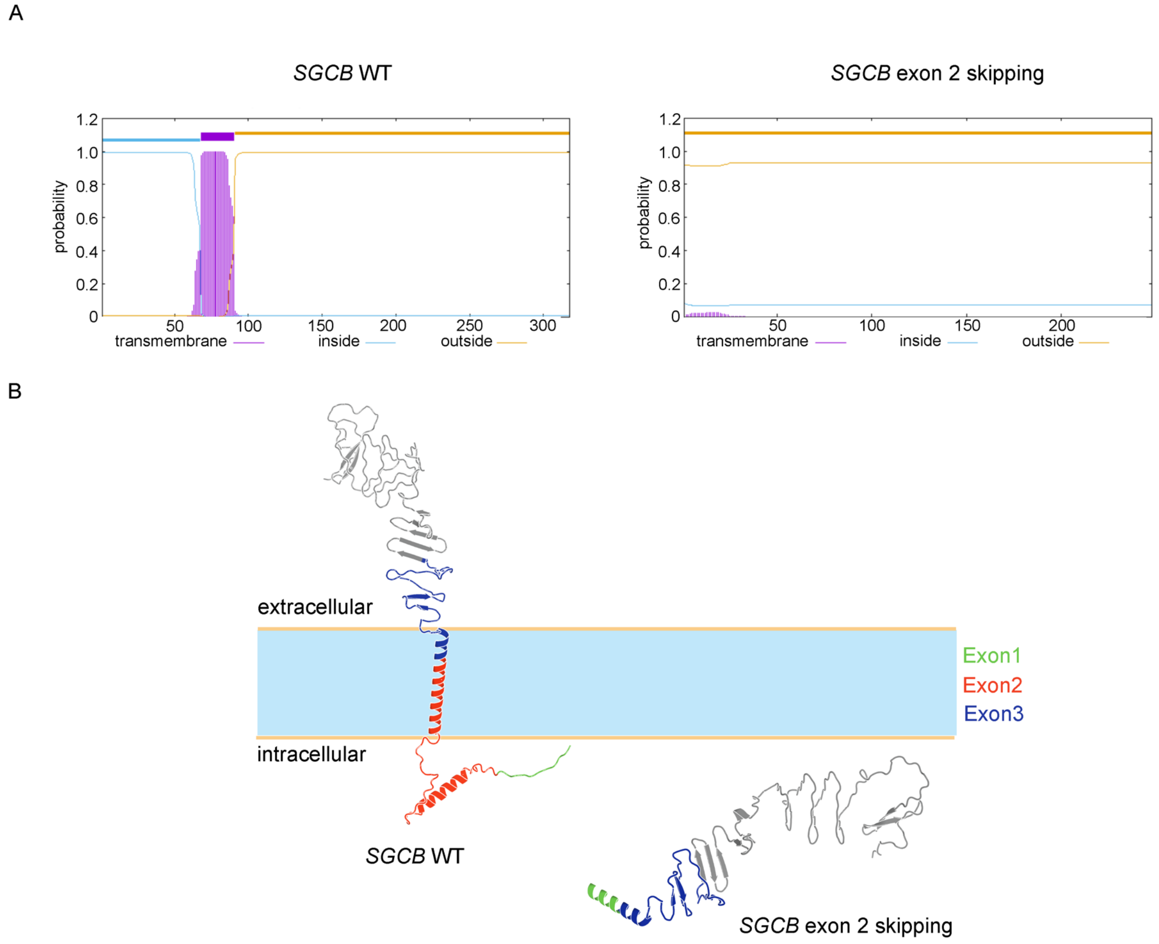

Supplemental Figure 7. Predicted structural consequences of *SGCB* exon 2 skipping. (A) TMHMM-based topology prediction of the transmembrane region in the wild-type (left) and exon 2–skipping (right) proteins. (B) Schematic comparison of the predicted membrane topology between the wild-type and exon 2–skipping proteins, based on AlphaFold structural predictions. The exon 2–skipping protein is predicted to lack a functional transmembrane anchor.

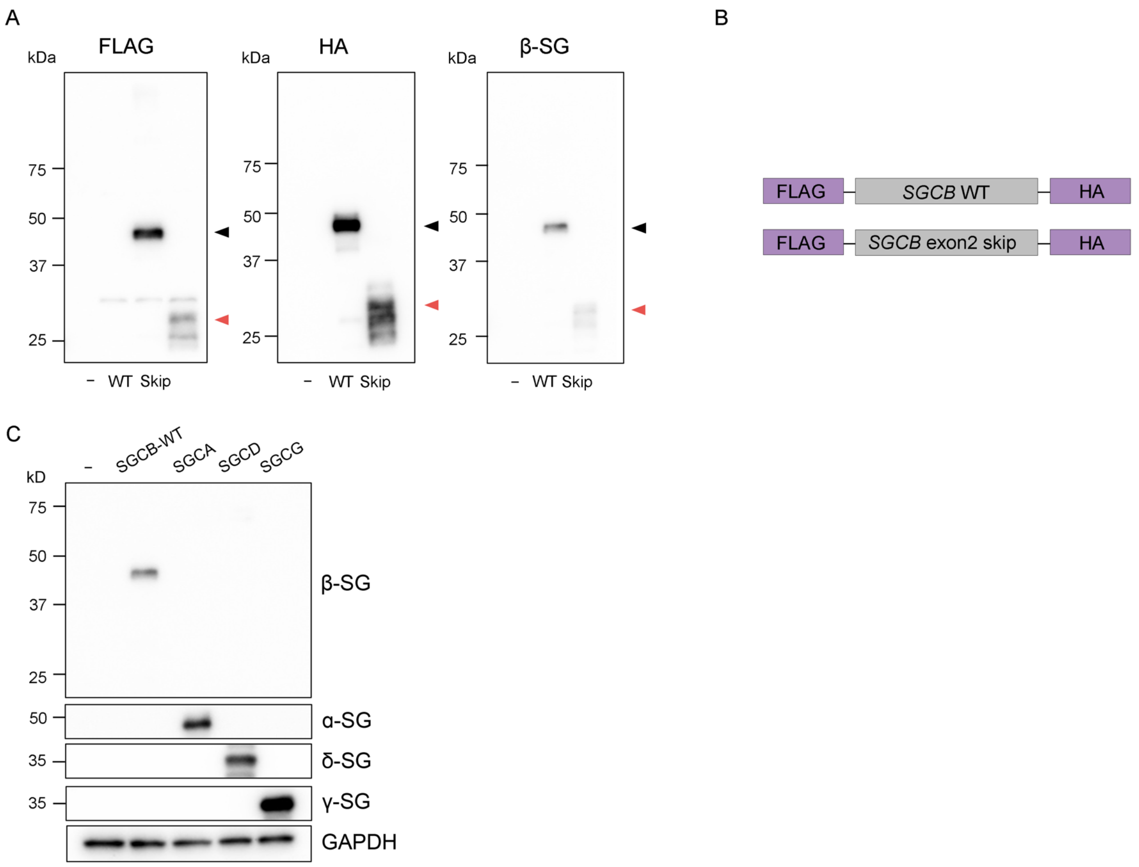

Supplemental Figure 8. Protein expression and antibody specificity analysis in 293T cells transfected with sarcoglycan constructs. (A) Western blotting of protein extracts from 293T cells using anti-FLAG, anti-HA, and anti-β-sarcoglycan (β-SG) antibodies. "–" indicates untransfected cells; "WT", cells transfected with the *SGCB* wild-type construct; "skip", cells transfected with the *SGCB* exon 2–skipping construct. (B) Schematic representation of the expression constructs used for transfection. Black arrows indicate the full-length *SGCB* protein; Red arrows indicate the exon 2–skipped isoform. (C) Western blotting of protein extracts from 293T cells to assess antibody specificity against individual sarcoglycan subunits, using anti-β-SG, anti-α-sarcoglycan (α-SG), anti-δ-sarcoglycan (δ-SG), and anti-γ-sarcoglycan (γ-SG) antibodies. "–" indicates untransfected cells; "*SGCB*-WT", cells transfected with the FLAG- and HA-tagged *SGCB* wild-type construct; "*SGCA*", "*SGCD*", and "*SGCG*", cells transfected with the respective untagged wild-type constructs.

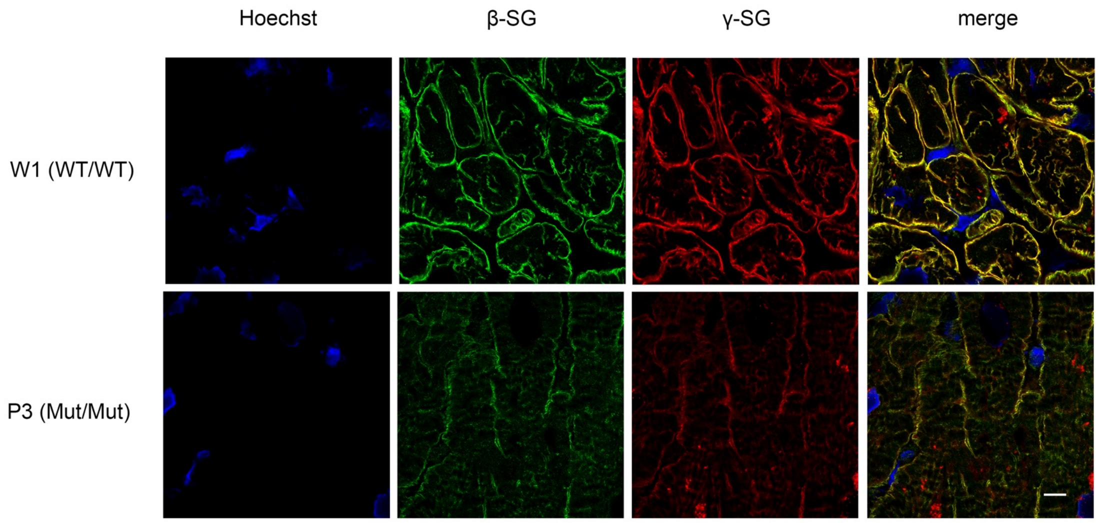

Supplemental Figure 9. Immunohistochemical co-staining of β-sarcoglycan (β-SG) and γ-sarcoglycan (γ-SG) in cardiac tissue. Representative images of cardiac sections from samples carrying the wild-type (WT/WT) or homozygous c.243+6T>A (Mut/Mut) allele of *SGCB*, stained with Hoechst (nuclei), anti-β-SG, and anti-γ-SG antibodies. The W1 (WT/WT) β-SG and P3 (Mut/Mut) β-SG panels are reproduced from Figure 4 to facilitate comparison of β-SG/ γ-SG colocalization. Images were acquired at ×1000 magnification. Scale bar: 20 μm.

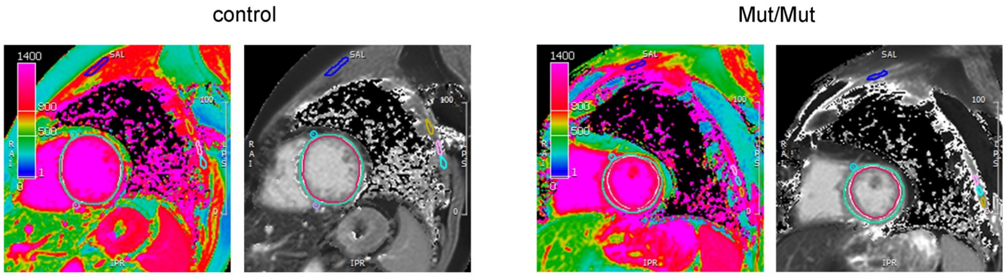

Supplemental Figure 10. Cardiac magnetic resonance T1 mapping of the heart, serratus anterior, and pectoralis major muscles. Representative short-axis native T1 maps from healthy controls and patients homozygous for the *SGCB* c.243+6T>A variant (Mut/Mut) are shown, with regions of interest (ROIs) used for T1 value calculation indicated.

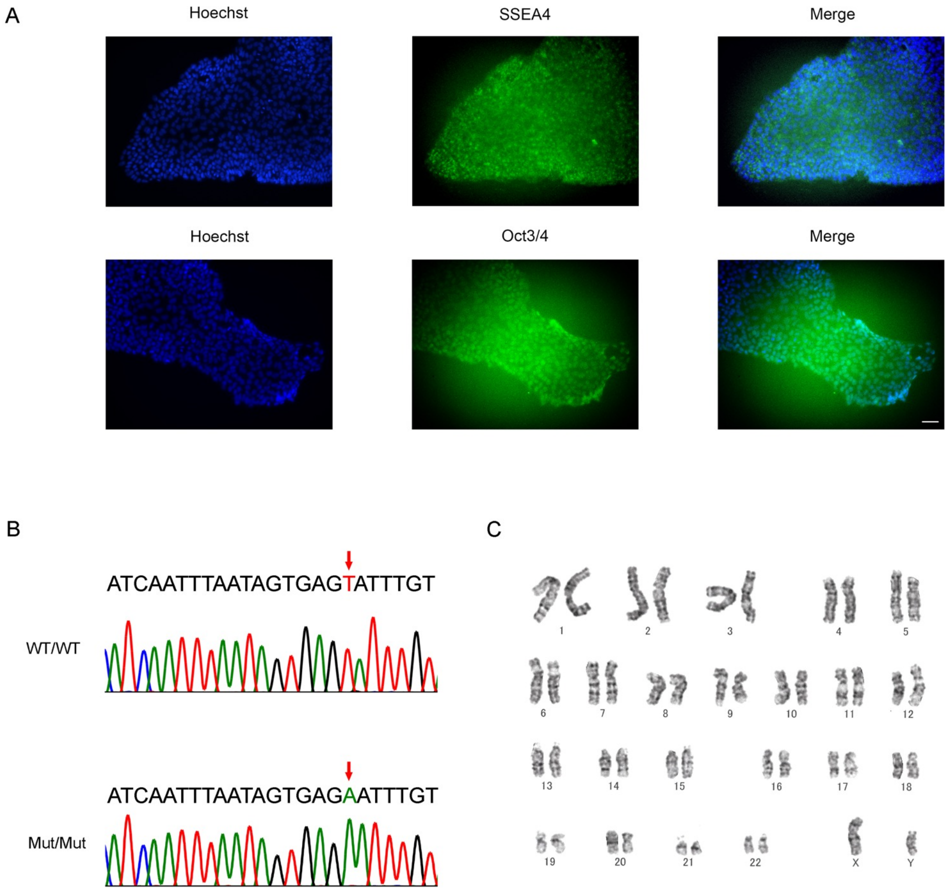

Supplemental Figure 11. Characterization of human induced pluripotent stem cells (hiPSCs) generated from a patient carrying the homozygous *SGCB* c.243+6T>A variant. (A) Immunofluorescence staining of hiPSCs using antibodies against pluripotency markers. Images were acquired at ×40 magnification. Scale bar: 50 µm. (B) Sanger sequencing of the *SGCB* locus in hiPSCs derived from a control line (253G1; WT/WT) and a patient carrying the homozygous *SGCB* c.243+6T>A variant (Mut/Mut). (C) Karyotype analysis of hiPSCs derived from the patient (Mut/Mut).

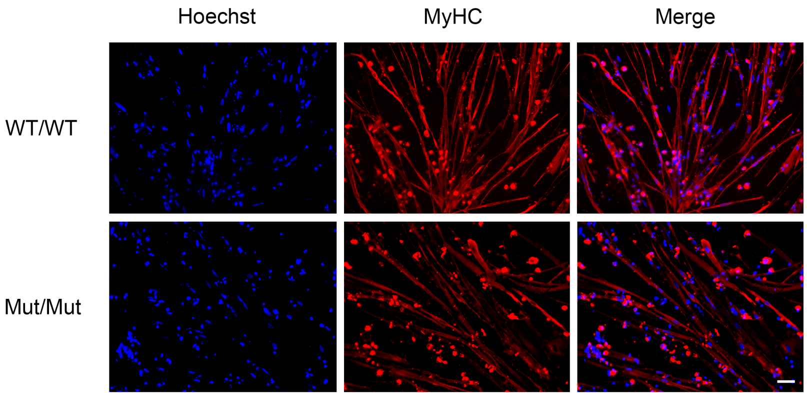

Supplemental Figure 12. Immunofluorescence staining of human induced pluripotent stem cell (hiPSC)–derived skeletal myocytes. hiPSCs derived from a control line (253G1; WT/WT) and a patient carrying the homozygous *SGCB* c.243+6T>A variant (Mut/Mut) were differentiated into skeletal myocytes and stained with an antibody against myosin heavy chain (MyHC). Images were acquired at ×40 magnification. Scale bar: 50 μm.

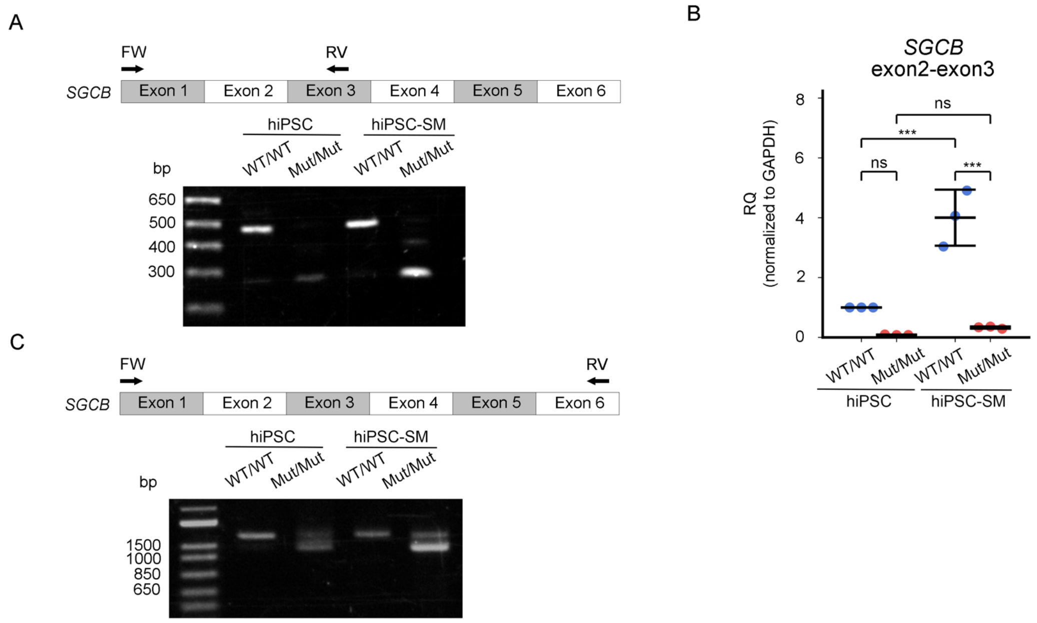

Supplemental Figure 13. RT-PCR and qPCR analysis of *SGCB* expression in human induced pluripotent stem cells (hiPSCs) and hiPSC-derived skeletal myocytes (hiPSC-SMs). (A) RT-PCR analysis of hiPSCs and hiPSC-SMs derived from a control line (253G1; WT/WT) and a patient carrying the homozygous *SGCB* c.243+6T>A variant (Mut/Mut), using primers spanning exon 1 to exon 3. (B) qPCR analysis of *SGCB* cDNA expression using a probe targeting the exon 2–3 junction in WT/WT and Mut/Mut hiPSCs and hiPSC-SMs. (C) RT-PCR analysis from hiPSCs and hiPSC-SMs from WT/WT and Mut/Mut lines, using primers spanning exon 1 to exon 6.

Gene Symbol			
ABCC9	*DSP*	*MYH6*	*RRAGD*
ACTC1	*EYA4*	*MYH7*	*SCN5A*
ACTN2	*FLNC*	*MYPN*	*SGCD*
ANKRD1	*ILK*	*NEBL*	*TCAP*
BAG3	*JPH2*	*NEXN*	*TNNC1*
BAG5	*LAMA4*	*PDLIM3*	*TNNI3*
CRYAB	*LDB3*	*PKP2*	*TNNT2*
CSRP3	*LMNA*	*PLN*	*TPM1*
DES	*LMOD2*	*PPCS*	*TTN*
DSG2	*MYBPC3*	*RBM20*	*VCL*

Supplemental Table 1. 40 DCM-associated genes included in variant screening

Patient ID	Age (y)	Sex	Disease	LVEF (%)	CK (U/L)*	Average Grip strength/BMI** (kg/BMI)	Average Knee Extension strength/BMI** (kgf/BMI)
W4	39	M	DCM	20	69	1.36 ± 0.15	2.12 ± 0.75
W5	52	M	DCM	27	147	1.27 ± 0.38	1.30 ± 0.06
W6	32	M	DCM	21	99	1.51 ± 0.09	2.14 ± 0.25
W7	58	M	DCM	20	47	1.93 ± 0.08	1.64 ± 0.13
P2	34	M	DCM	16	132	1.97 ± 0.04	1.97 ± 0.30
P3	50	M	DCM	17	130	1.67 ± 0.18	1.98 ± 0.39
P5	49	M	DCM	30	82	1.04 ± 0.18	1.71 ± 0.43
P7	38	M	DCM	24	187	1.41 ± 0.08	1.56 ± 0.13
P8	39	M	DCM	20	77	1.97 ± 0.17	1.74 ± 0.16

Supplemental Table 2. Muscle strength and related clinical parameters in patients harboring *TNNT2* or *SGCB* variants. Grip strength and knee extension strength were evaluated in patients harboring either the heterozygous *TNNT2* c.407G>A variant (W4, W5, W6, W7) or the homozygous *SGCB* c.243+6T>A variant (P2, P3, P5, P7, P8) (see also Supplemental Table 4). Strengths normalized to BMI are shown as mean ± SD. M, male; DCM, dilated cardiomyopathy; LVEF, left ventricular ejection fraction; CK, creatine kinase. *The normal reference range for serum CK is 54–286 U/L. **BMI was calculated as weight (kg) divided by height squared (m^2).

Patient ID	Sex	CK (U/L)
P1	Male	216
P2	Male	132
P3	Male	130
P4	Male	190
P5	Male	82
P6	Male	61
P7	Male	187
P8	Male	77
P9	Male	71
P10	Female	53
P11	Male	127
P12	Male	82

Supplemental Table 3. Serum creatine kinase (CK) levels in patients carrying the homozygous *SGCB* c.243+6T>A variant.

Characteristics	Control	*SGCB*	p-value
n	6	2*/3+	-
Age (yr)+	51.33 ± 10.88	61.00 ± 19.97	0.499
Myocardial global mean T1 (ms)*	966.50 ± 22.78	1017.50 ±10.61	0.011
Serratus anterior T1 (ms)+	893.44 ± 49.03	911.56 ± 85.70	0.759
Pectoralis major T1 (ms)+	887.83 ± 63.06	928.67 ± 11.37	0.179

Supplemental Table 4. Native T1 mapping values of the myocardium and skeletal muscles in healthy controls and patients homozygous for the *SGCB* c.243+6T>A variant. * n = 2 for myocardial T1 (one patient excluded due to prior heart transplantation); + n = 3 for skeletal muscle T1. Values are presented as mean ± SD.

Patient ID	Family	Age at Diagnosis (y)	Sex	Age at VAD (HTx) (y)	LVDd (mm)	LVEF (%)	Family History of CM
P1	-	47	M	59(61)	69	18	No
P2	Family 1: II5	30	M	30(34)	80	16	No
P3	-	50	M	50	81	17	No
P4	Family 4: II3	23	M	41(44)	84	17	No
P5	Family 3: II5	43	M	N/A	65	30	Yes
P6	Family 3: II4	39	M	N/A	67	32	Yes
P7	Family 2: II3	38	M	N/A	73	24	No
P8	-	26	M	39	73	20	No
P9	-	71	M	N/A	71	21	No
P10	-	47	F	63	66	33	No
P11	-	58	M	N/A	63	26	No
P12	-	35	M	N/A	89	19	No

Supplemental Table 5. Clinical characteristics of patients with dilated cardiomyopathy (DCM) homozygous for the c.243+6T>A variant in *SGCB*. M, male; F, female; VAD, ventricular assist device; HTx, heart transplantation; LVDd, left ventricular end-diastolic diameter; LVEF, left ventricular ejection fraction; CM, cardiomyopathy; N/A, not applicable (e.g., VAD or HTx not performed).

Primer	Sequence
Sanger primer FW1	ACAGTGGGACTGTTGAAAGCA
Sanger primer RV1	TGTGCATGGGTTTATCCCTTTA
Sanger primer FW2	ATGCACCAAAACGAGAGGGT
cDNA PCR primer FW	ACAGTCGGGCGGGGAGCTCGGC
cDNA PCR primer RV	GCTGGTTGTTGCCAGTGATGACCA

Supplemental Table 6. Primer sequences used for Sanger sequencing and cDNA PCR. "FW" and "RV" indicate forward and reverse primers, respectively.

Patient ID	Gene	Zygosity	Variant	Disease
W1	*PLN*	Het	c.40_42delAGA	DCM
W2	*TNNT2*	Het	c.452G>A	DCM
W3	*TNNT2*	Het	c.452G>A	DCM
W4	*TNNT2*	Het	c.452G>A	DCM
W5	*TNNT2*	Het	c.452G>A	DCM
W6	*TNNT2*	Het	c.452G>A	DCM
W7	*TNNT2*	Het	c.452G>A	DCM
P1	*SGCB*	Hom	c.243+6T>A	DCM
P2	*SGCB*	Hom	c.243+6T>A	DCM
P3	*SGCB*	Hom	c.243+6T>A	DCM
P4	*SGCB*	Het	c.243+6T>A, c.325C>T	DCM
P5	*SGCB*	Hom	c.243+6T>A	DCM
P6	*SGCB*	Hom	c.243+6T>A	DCM
P7	*SGCB*	Hom	c.243+6T>A	DCM
P8	*SGCB*	Hom	c.243+6T>A	DCM
P9	*SGCB*	Hom	c.243+6T>A	DCM
P10	*SGCB*	Hom	c.243+6T>A	DCM
P11	*SGCB*	Hom	c.243+6T>A	DCM
P12	*SGCB*	Hom	c.243+6T>A	DCM
H1	*SGCB*	Het	c.243+6T>A	RCM
H2	*SGCB*	Het	c.243+6T>A	Sarcoidosis
H3	*SGCB*	Het	c.243+6T>A	DCM
H3	*TTN*	Het	c.49870C>T	DCM

Supplemental Table 7 Summary of gene variants and clinical diagnoses for patients included in this study. Het, heterozygous; Hom, homozygous; DCM, dilated cardiomyopathy; RCM, restrictive cardiomyopathy.
