# Supplementary material for: Homozygous SGCB splice-site variant causes isolated dilated cardiomyopathy through sarcoglycan complex destabilization in East Asians
Source: J Clin Invest. 2026 Jun 1;136(11):e198675. doi: 10.1172/JCI198675 (PMC13221219; doi:10.1172/JCI198675)

Full unedited blot gel for Figure [3B]

β-SG

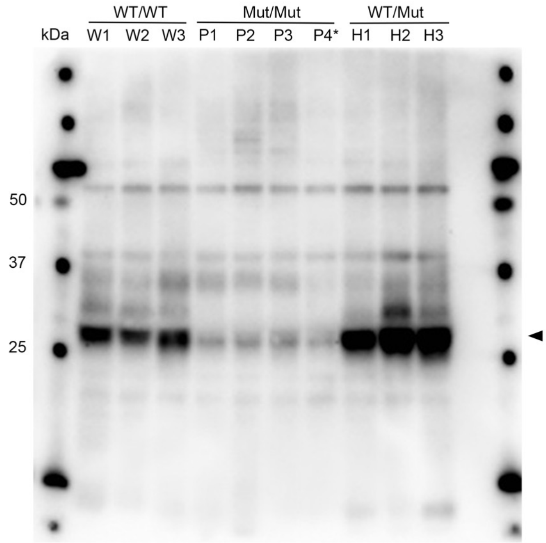

δ-SG

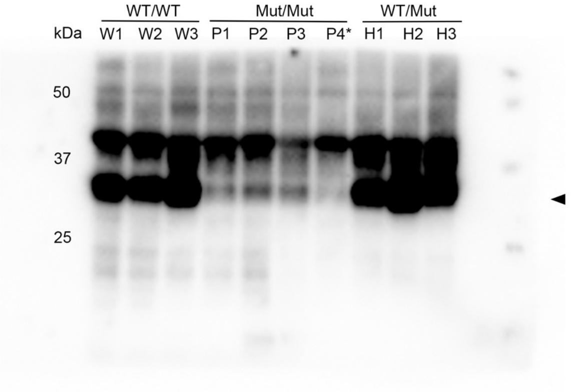

α-SG

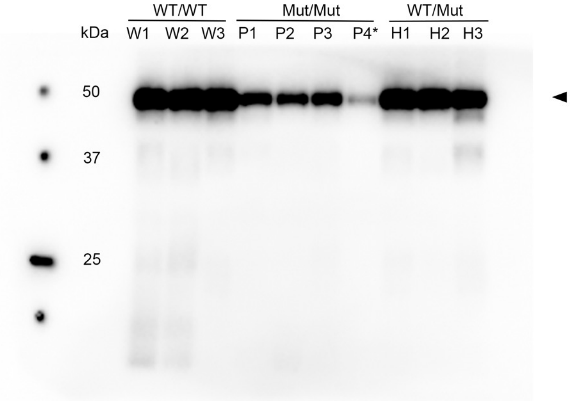

γ-SG

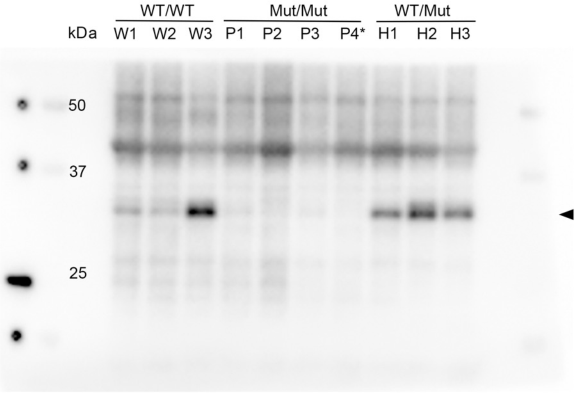

GAPDH

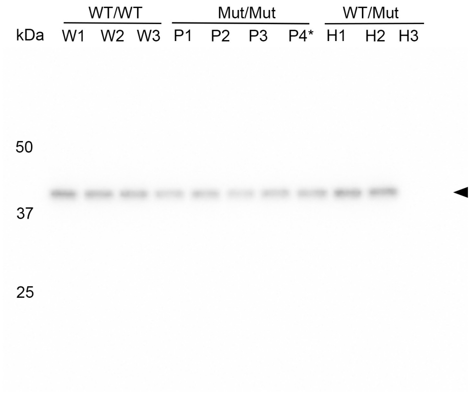

Ponceau S

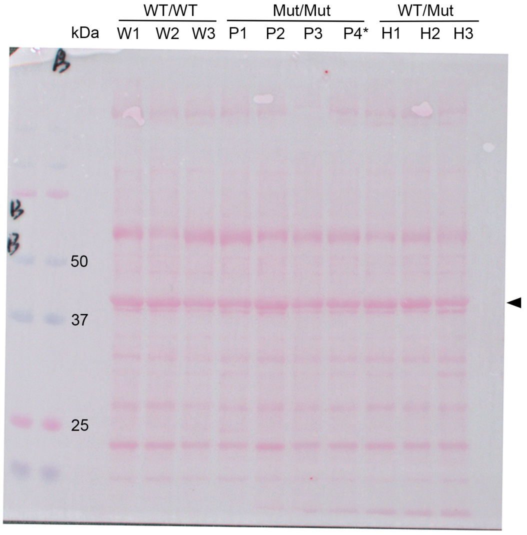

α-actinin 2

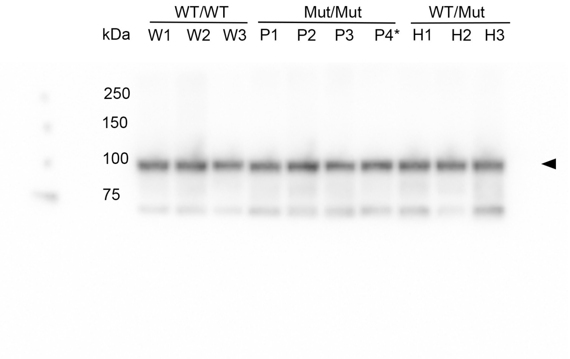

Full unedited blot gel for Figure [6B]

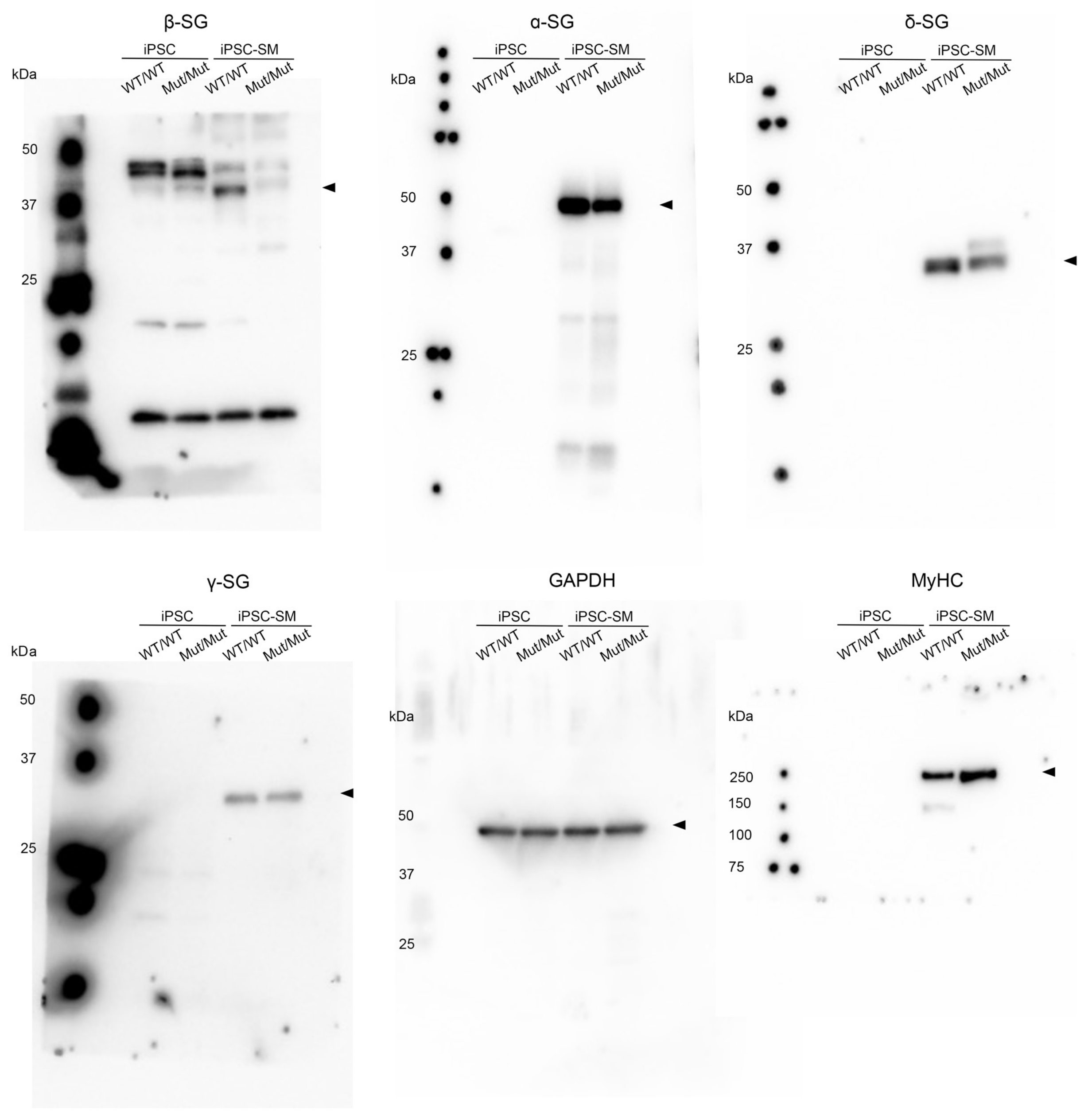

Full unedited blot gel for Figure [S3]

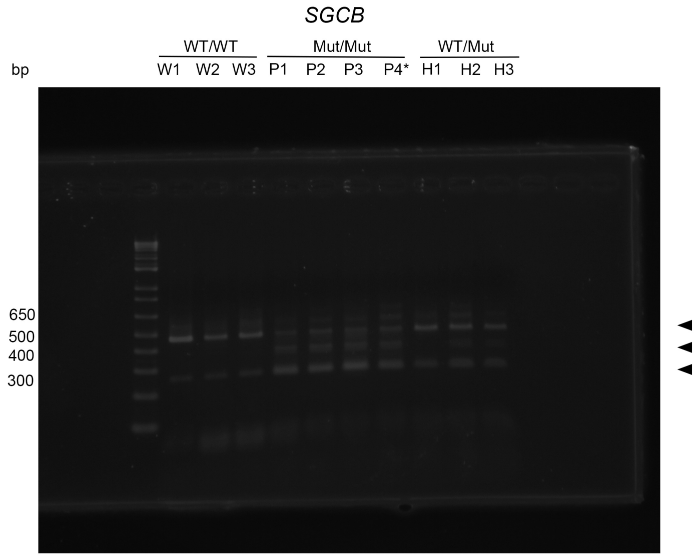

Full unedited blot gel for Supplemental Figure [S8A]

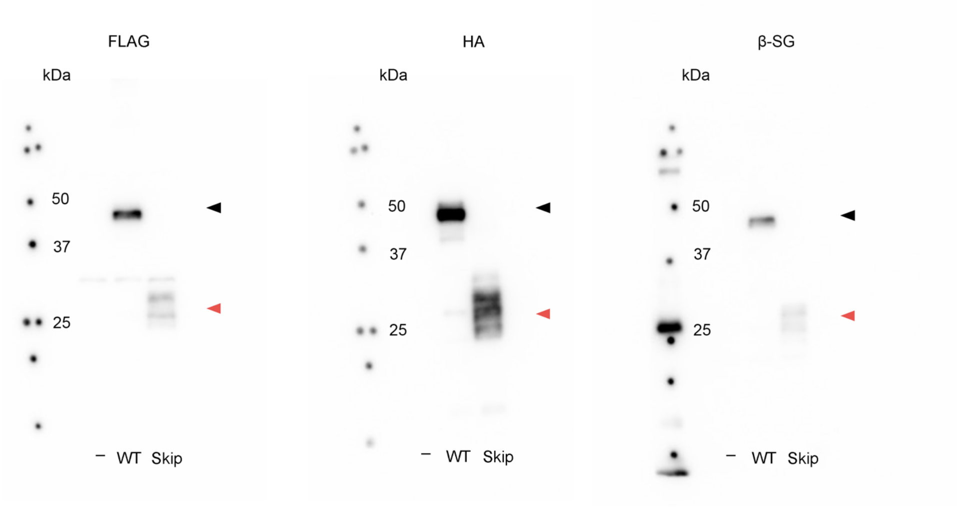

Full unedited blot gel for Supplemental Figure [S8C]

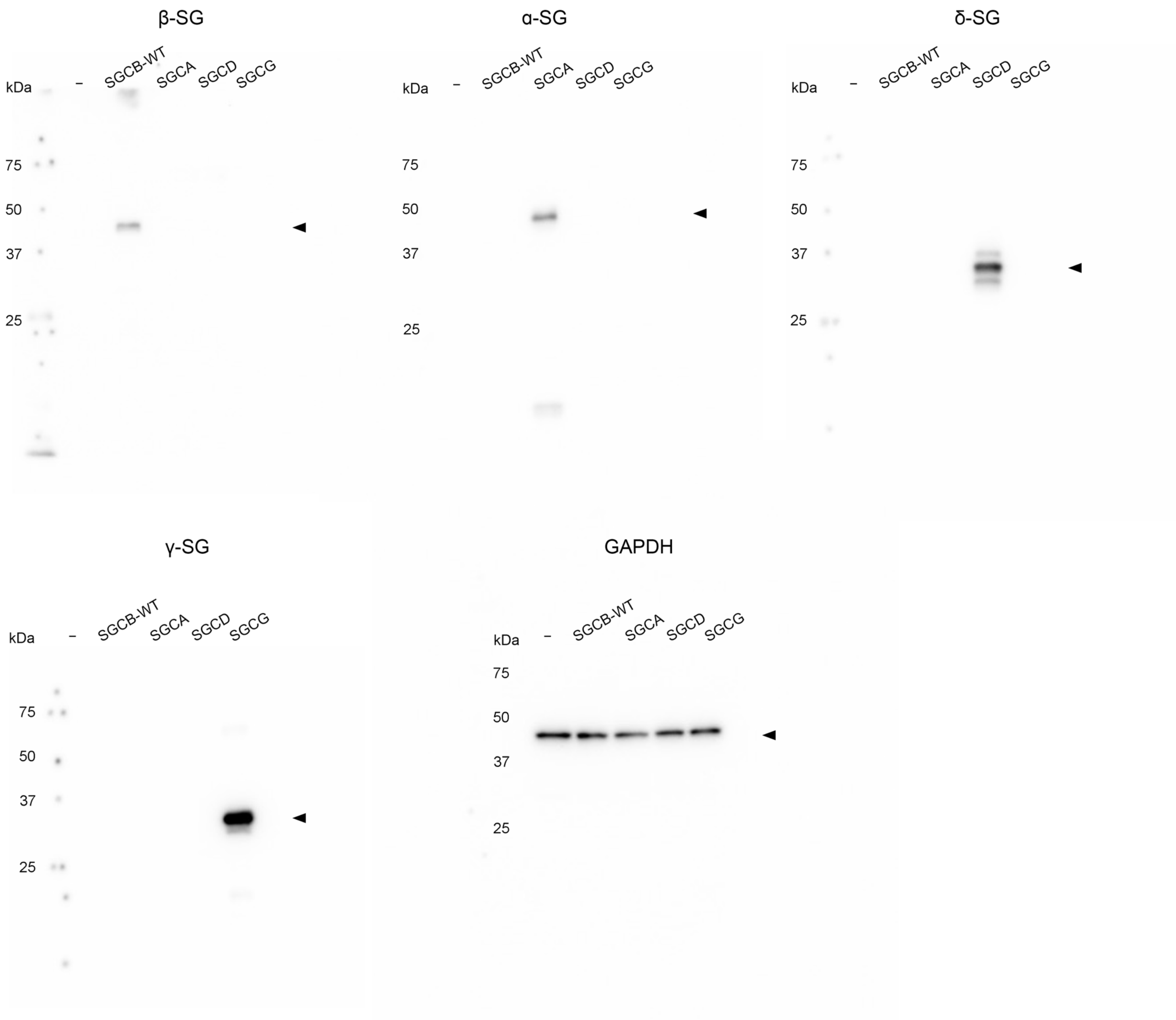

Full unedited blot gel for Figure [S13A]

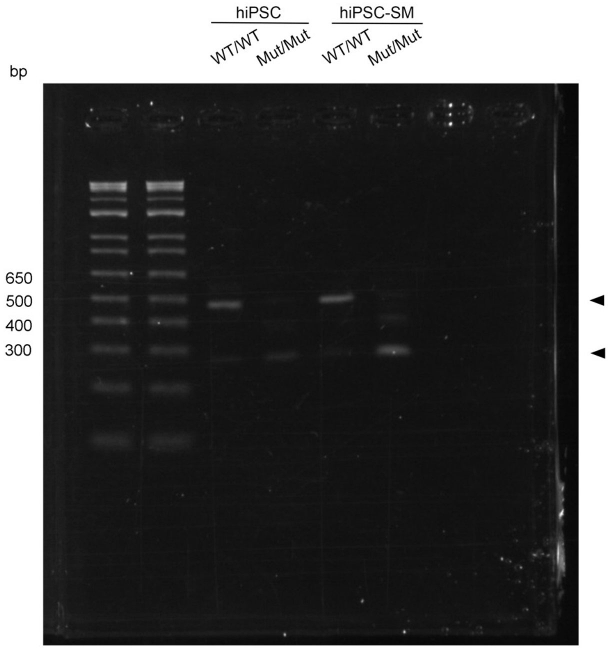

Full unedited blot gel for Figure [S13C]

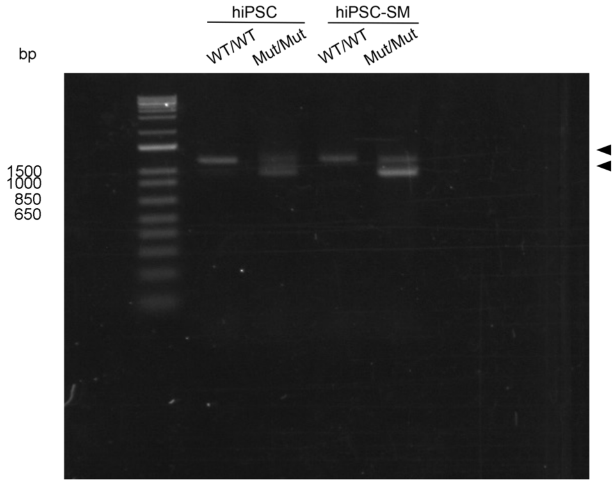

Supplement: Unedited blot and gel images [file jci-136-198675-s265.pdf]
